# The Protein Disulfide Isomerase gene family in bread wheat (*T. aestivum L*.)

**DOI:** 10.1186/1471-2229-10-101

**Published:** 2010-06-03

**Authors:** Elisa d'Aloisio, Anna R Paolacci, Arun P Dhanapal, Oronzo A Tanzarella, Enrico Porceddu, Mario Ciaffi

**Affiliations:** 1 Scuola Superiore Sant'Anna, Piazza Martiri della Libertà 33, 56127 Pisa, Italy; 2 Dipartimento di Agrobiologia e Agrochimica, Università della Tuscia, Via S. Camillo De Lellis, 01100 Viterbo, Italy

## Abstract

**Background:**

The Protein Disulfide Isomerase (PDI) gene family encodes several PDI and PDI-like proteins containing thioredoxin domains and controlling diversified metabolic functions, including disulfide bond formation and isomerisation during protein folding. Genomic, cDNA and promoter sequences of the three homoeologous wheat genes encoding the "typical" PDI had been cloned and characterized in a previous work. The purpose of present research was the cloning and characterization of the complete set of genes encoding PDI and PDI like proteins in bread wheat (*Triticum aestivum *cv Chinese Spring) and the comparison of their sequence, structure and expression with homologous genes from other plant species.

**Results:**

Eight new non-homoeologous wheat genes were cloned and characterized. The nine PDI and PDI-like sequences of wheat were located in chromosome regions syntenic to those in rice and assigned to eight plant phylogenetic groups. The nine wheat genes differed in their sequences, genomic organization as well as in the domain composition and architecture of their deduced proteins; conversely each of them showed high structural conservation with genes from other plant species in the same phylogenetic group. The extensive quantitative RT-PCR analysis of the nine genes in a set of 23 wheat samples, including tissues and developmental stages, showed their constitutive, even though highly variable expression.

**Conclusions:**

The nine wheat genes showed high diversity, while the members of each phylogenetic group were highly conserved even between taxonomically distant plant species like the moss *Physcomitrella patens*. Although constitutively expressed the nine wheat genes were characterized by different expression profiles reflecting their different genomic organization, protein domain architecture and probably promoter sequences; the high conservation among species indicated the ancient origin and diversification of the still evolving gene family. The comprehensive structural and expression characterization of the complete set of *PDI *and *PDI*-like wheat genes represents a basis for the functional characterization of this gene family in the hexaploid context of bread wheat.

## Background

Thousands of secretory proteins are distributed into the different compartments of the endomembrane system after their synthesis in the endoplasmic reticulum (ER), where resident proteins assist their folding and assembly and monitor their correct three-dimensional conformation, sorting for disposal those that fail to fold or to assemble properly [1,2]. Disulfide bonds cross-linking specific cysteines are formed during the maturation of secretory proteins to stabilize them and to join covalently multiple subunits; mispairing of cysteine residues can lead to misfolding [3]. Lines of evidence support the catalytic role of protein disulfide isomerase (PDI) and PDI-related proteins of the thioredoxin (TRX) superfamily in the correct formation of disulfide bonds in nascent polypeptides. The TRX proteins are characterized by the presence of one or more "TRX-like" domains showing sequence or structural similarity to the thioredoxin active site [4].

The classical or typical PDI (EC 5.3.4.1) is one of the most abundant proteins in the ER; it is a homodimer formed by two subunits of about 57 kDa, whose structure and function have been extensively studied in mammalian species, particularly in humans [reviewed in [5]]. It catalyzes thiol-disulfide oxidation, reduction and isomerisation, this latter occurring directly by intramolecular disulfide rearrangement or through cycles of reduction and oxidation [6]. The ability of PDI to bind to unfolded or partially folded proteins preventing their aggregation has also suggested its role as a chaperone [7], involved in the quality control system checking the correct folding of the proteins synthesized in the ER [8,9]. Two additional features as subunit of complex enzymatic systems have been demonstrated in mammal PDI is the β-subunit of prolyl 4-hydroxylase [10] and it is a component of the triglyceride transfer complex [11]. Human PDI has a modular structure including four TRX-like domains (***a***, ***b***, ***b' ***and ***a'***), a linker (x) and a C-terminal extension domain (***c***) [for reviews see [12,13]]. The ***a ***and ***a' ***domains are homologous to thioredoxin and contain a catalytic site for isomerase and redox activities consisting of the Cys-Gly-His-Cys amino acid sequence [14], whereas the middle ***b ***and ***b' ***retain only similarities to the TRX domain within their secondary structure [15]. The ***c ***domain at the C terminal region, is rich of acidic residues typical of calcium binding proteins [16] and ends with a KDEL sequence for ER retention [17].

The typical PDI is the most prominent member of a family of related PDI-like proteins characterised by the presence of one to three thioredoxin-like active domains [5]. Several genes encoding PDI-like proteins with unusual primary structure, different expression pattern and exhibiting a wide range of activities have been identified in every extensively sequenced mammalian genome [5,8,18,19]. Even though all proteins of the PDI family have been located within the ER, where they perform their function, Turano et al. [8] have located some of them in different subcellular locations, but their export mechanism is still unknown.

In higher plants, 13 genes have been identified in Arabidopsis (*Arabidopsis thaliana*) and 12 in rice (*Oryza sativa*) and in maize (*Zea mays*) [20]. Phylogenetic analyses of their protein sequences have shown that the plant PDI family would include at least eight different subfamilies. The proteins of the first five groups or subfamilies (I-V) have two thioredoxin-like active domains and show structural similarity to other PDI-like proteins of higher eukaryotes, whereas the proteins of the remaining three subfamilies (VI-VIII) contain a single thioredoxin-like active domain. The first group (I) includes the typical PDI, which has been cloned and sequenced in several plant species.

In cereals most studies on molecular characterization, transcriptional regulation and intracellular localization of genes and proteins of the PDI family have concerned the typical PDI [21,22], which may accomplish an important role in the folding of plant secretory proteins, particularly during the formation of endosperm protein bodies. The importance of PDI in the storage protein deposition in cereals is also supported by the analysis of some maize and rice mutants, which form seeds with altered endosperm protein bodies [23,24]; the lack of PDI expression caused failure in the correct formation of protein bodies, suggesting its essential and direct role in their formation.

Genes coding for novel PDI family proteins of groups I, II, III, IV and V have been cloned in soybean [25-28]; they are ubiquitously expressed and their encoded proteins located in the ER. The proteins encoded by genes of the groups I, II, IV and V contain two standard -CGHC- catalytic sites and have thiol oxidoreductase activity *in vitro*. They seem to be involved in the folding of storage proteins in cotyledons, such as the proglycinin, a precursor of the seed storage protein glycinin, and the β-conglycinin; both acting as thiol-oxidoreductase and as a molecular chaperone. The proteins encoded by the genes of the III group contain non standard -CXXS/C redox active sites and do not exhibit oxidoreductases or molecular chaperone activities *in vitro*, consequently they may be involved in the maturation of seed cotyledons through molecular mechanisms differing from the other PDI family members.

PDI-like proteins would also be involved in signal transduction pathways through their association with transcriptional complexes regulating genes responding to various stimuli. In *Chlamydomonas reinhardtii *the PDI-like protein RB60 is involved in the maintenance of photosynthetic capacity as part of a redox regulatory protein complex controlling translation in the chloroplast [29]; RB60 resides also in the ER, wherein its function is unknown [30].

The involvement of the typical PDI and probably of additional PDI-like proteins in the folding of endosperm storage proteins is especially important in wheat, wherein the processes occurring during protein synthesis and deposition may affect the functional properties of gluten. Wheat storage proteins consist primarily of prolamins synthesised in the developing endosperm and targeted to the ER lumen, where they are folded and connected by intermolecular disulfide bonds to form large aggregates [31]. The storage proteins play an integral role in determining the visco-elastic properties of wheat dough, with larger polymers being related to increased elasticity, a feature of high quality wheat [32]. Therefore, the genes encoding storage proteins, as well as factors that may affect their deposition, such as molecular chaperones and foldase enzymes, are of particular interest to wheat industry. Even though wheat storage proteins have been the object of a wide range of studies both at chemical and genetic levels [reviewed in [31,32]], the knowledge of factors affecting their folding and deposition is still extremely limited.

Considering potential applications to the improvement of flour quality, we have undertaken a research programme on the molecular characterization of the PDI gene family in wheat. The three homoeologous genes coding for the typical PDI and their promoter sequences had previously been isolated and characterized in common wheat [33]. Their exon/intron structure, whose sequences have been located in chromosome arms 4AL, 4BS and 4DS of hexaploid wheat [34], is highly conserved and includes 10 exons. Expression analysis has shown that transcripts of the typical PDI, though constitutively present at a low level in all the analyzed tissues, are particularly abundant in the developing caryopses [33,35]. The detection, within the upstream putative promoter region, of several cis-acting elements involved in endosperm specific expression is consistent with the higher PDI transcript expression detected in kernels. In this paper we report the isolation and characterization of the complete set of PDI related gene sequences of wheat, which, in addition to the typical PDI, include eight new non-homoeologous genes coding for PDI-like proteins. Moreover, the paper reports their assignment to the eight phylogenetic groups of the plant PDI family, their chromosome location, the organization of their genomic sequences and their expression profiles in a set of 23 samples, including different tissues and developmental stages.

## Methods

### Plant material

The following tissue samples were collected (January-June 2008) from 20 bread wheat plants (*Triticum aestivum *cv Chinese Spring) grown in open field at Viterbo (Italy), immediately frozen in liquid nitrogen and kept at -80°C until use for RNA isolation: 1) roots from plants with single shoot and three leaves unfolded (Feekes scale 1.3); 2) the above-ground portion from the same plants; 3) shoots at the beginning of tillering (Feekes scale 2.0); 4) shoots from plants with formed tillers (Feekes scale 3); 5) shoots at the beginning of erect growth (Feekes scale 4); 6) stems at booting stage (Feekes scale 10); 7) flag leaves at booting stage (Feekes scale 10); 8) spikes collected at intervals of 10 to12 days (three developmental stages: 15-20 mm, flag leaf unfolding and heading stage); 9) single floral organs (glumes, palea, lemma, lodicules, stamens and pistil) from fully emerged spikes (Feekes scale 10.5); 10) developing caryopses from 5 to 38 days after anthesis (DAA) at 5 to 6 days intervals (7 samplings).

### DNA and RNA isolation

DNA was isolated from 5 g of leaves collected from single plants of *Triticum aestivum *cv Chinese Spring (CS) and its nulli-tetrasomic (NT) lines as reported in [34]. Total RNA was extracted using the TRIzol reagent (Invitrogen) according to manufacturer's instructions, whereas from caryopses it was isolated by a LiCl based method [35]. RNA concentration and quality integrity were checked as described in [36].

### Identification and amplification of full length wheat PDI-like cDNAs

The available sequences of PDI-like genes of rice (12 sequences) and Arabidopsis (13 sequences) [20] were exploited to BLAST search three public databases of wheat ESTs (Expressed Sequence Tags): DFCI wheat gene index database (TaGI, version 11), HarvEST wheat (version 1.13) and NCBI. BLAST searches identified eight novel non-redundant PDI-like consensus sequences, which were used as templates for 5' and 3' RACE (Rapid Amplification of cDNA Ends) extensions using the 5'/3' RACE kit from ROCHE following manufacturer's instructions. RACE products were amplified (sequences of RACE primers are available upon request) using 2 μg of a pool of total RNA from different CS tissues (see Plant material). The 5' and 3' RACE products were cloned and validated by sequence analysis; the corresponding full-length cDNAs of eight novel wheat PDI-like genes were cloned by RT-PCR using total RNA from different tissues of CS and specific primers designed on the basis of the 5' and 3' untranslated regions (see Additional file 1). First-strand cDNA was synthesized from 3 μg of RNA by the Expand™ Reverse Transcriptase (ROCHE) and the PCR reactions were performed using 2 μl of the RT reaction with the GC-Rich PCR System from ROCHE following manufacturer's instructions.

### Isolation of genomic sequences

The genomic sequences of the eight novel PDI-like genes of wheat were amplified using two different methods. Amplicons up to 5 kb were amplified using the GC-Rich PCR System from ROCHE, with the following modification to the method used for cDNA synthesis: 400 ng of genomic DNA as template; for the first 10 cycles elongation of 5 to 8 minutes (depending on template size) at 66°C temperature, then increase of 5 s for each successive cycle; final elongation at 66°C for 7 minutes. PCR of the amplicons exceeding 5 kb was performed by the Expand Long Template PCR System (ROCHE) as directed in the package insert, using the supplied buffer 3 and 400 ng of genomic DNA. DNA was denatured at 94°C for 2 min, then amplified by 10 cycles each at 94°C for 30 s, 58 to 65°C (depending on the primer) for 30 s and 68°C for up to 15 min, followed by additional 25 cycles, identical to the previous 10 except the elongation time was further extended by additional 20 s for each successive cycle, then a final elongation step at 68°C for 15 min was performed. Genomic sequences were amplified using the same combination of primer pairs employed for the cloning of the full-length cDNAs (Additional file 1).

### Cloning and sequencing of RACE, cDNA and genomic DNA amplification products

Amplification products of RACE, full-length cDNAs and genomic sequences were visualized on 1.2% agarose gel stained with ethidium bromide. PCR products of expected size were excised from the gel, purified using the High Pure Purification kit (ROCHE) according to manufacturer's instructions, and cloned into the pGEM-T easy plasmid vector (PROMEGA). Two independent PCR amplifications were performed for each cDNA, genomic and RACE amplicon, their products were cloned and for each reaction multiple clones were sequenced (6 cDNA, 2 genomic and 10 RACE clones). Plasmid DNA for sequencing reaction was prepared from 3 ml overnight cultures using a plasmid mini-prep kit (QIAGEN). Sequencing was performed on both strands by the ABI PRISM 377 capillary sequencer (PE Applied Biosystem) using an ABI Prism Dye Terminator sequencing kit (PE Applied Biosystem) and either vector or sequence specific primers. The complete sequences of the genomic clones were obtained by sequencing them with internal primers complementary to the cDNA sequences and designed near the predicted exon/intron junctions so to amplify each exon and nearby intron on both strands (primers are available upon request). All sequences were analyzed by DNAMAN Sequence Analysis Software (Version 3, Lynnon Biosoft) and their homologies were scored using the BLASTX program [37] through the National Center for Biotechnology Information (NCBI) GeneBank database [38]. The software developed by Hesbsgaard et al. [39] was used for the prediction of intron splice sites within the genomic sequences. Full-length cDNA and genomic sequences were deposited in the DDBJ/EMBL/GeneBank nucleotide sequence databases, accession numbers are indicated in Additional file 2. A code of two letters (Ta = *Triticum aestivum*) followed by the suffix PDIL and by an Arabic number indicating the corresponding phylogenetic group was assigned to each sequence. Multiple sequences clustering into the same subfamily were designed by an additional number (1-2). The predicted protein sequences were analysed by searching for conserved motifs in CDD [40], Pfam HMMs [41], InterPro [42] and SMART [43] databases; their subcellular locations were predicted by Target P1.1 [44] and ChloroP 1.1 [45], the presence of the signal peptide was confirmed by Signal P3.0 [46] and the transmembrane regions were determined by TMHMM ver 2.0 [47]. Protein identity was determined using the "two sequence alignment" option of DNAMAN software (Lynnon BioSoft, Canada) with the following settings: gap open penalty 8 and gap extension penalty 2 and BLOSUM protein weight matrix.

### Phylogenetic analysis

In addition to the eight novel wheat PDI-like sequences cloned in this study and the typical PDI isolated previously [33], 100 PDI and PDI-like sequences from Arabidopsis, rice, maize, soybean (*Glycine max*), grapevine (*Vitis vinifera*), poplar (*Populus trichocarpa*), *Physcomitrella patens *and *Chlamydomonas reinhardtii *were retrieved from published [20,25-28] and deposited [38,48-51] sequences. The nomenclature of PDI-like sequences of different plant species and of the moss *P. patens *was identical to that adopted for wheat (see Additional file 3). The full nomenclature included two letters for genus and species, followed by PDIL and by an Arabic number indicating the corresponding phylogenetic group; multiple sequences clustering into the same subfamily were designed by an additional number (1-3). The original nomenclature of the deposited sequences was adopted only for *C. reinhardtii*. Deduced amino acid sequences of the whole coding regions were aligned by ClustalX version 1.83 [52] using the Gonnet series as protein weight matrix and parameters set to 3 gap open penalty, 1.6 gap extension penalty, negative matrix on and divergent sequences delay at 36%. The phylogenetic tree was constructed using the neighbour-joining (NJ) method [53], as provided by the program NEIGHBOR of the PHYLIP package version 3.6. For tree reconstructions, distance matrices were estimated by the PHYLIP program PRODIST using the PAM model of amino acid transition. To evaluate statistical significance of the phylogenetic trees 1,000 bootstrap replicates were generated from each data set using the PHYLIP program SEQBOOT.

### Chromosome location

For Southern analysis genomic DNA (10 μg) from CS and its NT lines was digested with *Eco*RI, *Bam*HI, *Hin*dIII, *Sph*I, *Sac*I and *Dra*I, electrophoresed in 1% agarose gels, transferred to a positively charged nylon membrane (ROCHE) and hybridised with digoxigenin-labelled probes using the PCR-DIG Probe Synthesis Kit (ROCHE). The eight primer pairs used for probe labelling by PCR are reported in Additional file 4. Pre-hybridisation, hybridisation, washing and immunological detection were performed as reported in [34].

The hybridisation pattern of *TaPDIL2-1 *was complex, thus the chromosome locations of its homoeologous sequences were determined by PCR analysis with a primer pair (5'-CGTCAAAGTTGTTGTTGGCAA-3' and 5'-CCTACAACTCGTCCTTGGG-3') flanking a region spanning the last 3 introns of *TaPDIL2-1*. PCR reactions were performed with GoTaq DNA Polymerase (PROMEGA) following manufacturer's instructions and using 50 ng of genomic DNA from CS and its NT lines. Amplification products were analysed on a 2% agarose gel run at 40 V overnight and verified through sequence analysis.

### Expression analysis

The expression patterns of nine non-homoeologous genes coding for wheat PDI and PDI-like proteins were analysed by quantitative real time RT-PCR (qRT-PCR) in a set of 23 samples which included different tissues and developmental stages, as specified in Plant material. Quantitative RT-PCR analyses and data normalization were performed according to Paolacci et al. [36] and described in detail in the Additional file 5. Two biological replicates, resulting from two different RNA extractions, RT and qRT-PCR reactions, were used in quantification analysis; three technical replicates were analysed for each biological replicate.

The relative and absolute expression levels of the nine genes were computed considering four data sets, obtained by the different groupings of the analysed samples: 1) twelve samples relative to different tissues and developmental stages (roots, shoots, stems, leaves, spikes and caryopses at different developmental stages); 2) ten samples including the above data set except caryopses; 3) six samples represented by single floral organs from fully emerged spikes; 4) seven samples relative to developing caryopses collected between 5 and 38 DPA.

## Results and Discussion

### Cloning and characterization of PDI-like genes in wheat

The identification of cDNA sequences of wheat PDI-like genes was based on the BLAST search of the DFCI Wheat Gene Index (TaGI, version 11) [54] using the available PDI-like gene sequences of rice (*Oryza sativa*, 12 sequences) and Arabidopsis (*Arabidopsis thaliana*, 13 sequences) [20]. The TaGI search by BLAST fetched nine distinct contigs (tentative consensus sequences), one of them (TC310491) encoded the typical PDI, whose three homoeologous genes had previously been cloned and characterised [33]. An additional search in HarvEST wheat, version 1.13 [55] and in all NCBI cDNA libraries of wheat detected several ESTs (Expressed Sequence Tags) homologous to rice and Arabidopsis PDI-like genes; ninety sequences were randomly selected among those exceeding 350 bp and were cloned by RT-PCR using an RNA mixture from different bread wheat cv CS tissues as template. On the basis of their homology the 90 cDNAs formed nine groups, corresponding to the tentative consensus (TC) sequences assembled from the ESTs detected in the TaGI database. The eight TC sequences (Table 1) corresponding to new wheat PDI-like genes were used as template to isolate by RACE the corresponding 5' and 3' extensions, subsequently validated by sequence analysis. Full-length cDNAs of the eight PDI-like genes (Table 1) were cloned by RT-PCR of RNA from various wheat tissues using specific primer pairs designed in the 5' and 3' UTRs (untranslated regions) (see Additional file 1). Two independent RT-PCR reactions were performed for each sequence; both amplified products had the same electrophoretic mobility and for each of them 3 clones were sequenced. For each of three full length PDI-like cDNAs (TaPDIL2-1, TaPDIL3-1 and TaPDIL8-1) all the six clones (3 clones × 2 RT-PCR reactions) exhibited identical sequences, whereas two slightly different sequences were recognized among the six clones analysed for each of four cDNA sequences (TaPDIL4-1, TaPDIL5-1, TaPDIL6-1, TaPDIL7-2) and three among the six clones of the remaining cDNA (TaPDIL7-1). The different clones obtained by RT-PCR using the same primer pair were identified by an additional letter (a, b and c) (Table 1). The deduced amino acid sequences of the 14 cloned cDNAs shared over 70% identity with those of the corresponding orthologous PDI-like genes of rice, whose nucleotide sequences had been used for the identification of the wheat ESTs (Table 1). Multiple cDNAs cloned from independent amplifications with the same primer pair showed high identity (over 96%) of both nucleotide and amino acid sequences (Table 2), most probably because they derived from transcripts of multiple copies of PDI-like genes located in homoeologous chromosomes of hexaploid wheat (AABBDD genome). Mutations detected in the coding region of the putative homoeologous cDNA sequences consisted mainly of nucleotide substitutions, most of them synonymous (Table 2). The most remarkable differences between putative homoeologous sequences consisted of three short in-frame insertion/deletion (indel), one of six nucleotides in the N-terminal region of TaPDIL6-1a and TaPDIL6-1b and two of three and nine nucleotides in the N-terminal region of TaPDIL7-1a, TaPDIL7-1b and TaPDIL7-1c (Table 2). The 5' and 3' UTRs showed slightly higher rates of base substitutions and of indels of variable length (data not shown). These data indicate that the negligible differences observed between the deduced amino acid sequences of the wheat putative homoeologous PDI-like genes would not justify their functional diversification.

**Table 1:** Characteristics of the full-length cDNA sequences coding for wheat PDI-like proteins cloned in this study.

Clone	Full length cDNA			TC sequence	Orthologous rice gene			Protein identity
	UTR5' (nt)	UTR3' (nt)	ORF (nt)	(DFCI wheat Gene index)	Previous name*	This study	Acc. number cDNA	
*TaPDIL2-1*	63	109	1767	TC301880	*OsPDIL1-4*	*OsPDIL2-1*	AK071514	408/561 (72.73%)
*TaPDIL3-1*	141	110	1626	TC353685	*OsPDIL1-5*	*OsPDIL3-1*	AK073970	437/529 (82.61%)
*TaPDIL4-1a*	74	139	1104	TC300461	*OsPDIL2-1*	*OsPDIL4-1*	AK103944	316/366 (86.34%)
*TaPDIL4-1b*	74	138	1104	TC300461	*OsPDIL2-1*	*OsPDIL4-1*	AK103944	317/366 (86.61%)
*TaPDIL5-1a*	55	150	1323	TC317379	*OsPDIL2-3*	*OsPDIL5-1*	AK062254	391/439 (89.07%)
*TaPDIL5-1b*	55	151	1323	TC317379	*OsPDIL2-3*	*OsPDIL5-1*	AK062254	397/439 (90.43%)
*TaPDIL6-1a*	13	240	456	TC294820	*OsPDIL5-1*	*OsPDIL6-1*	AK063663	121/146 (82.88%)
*TaPDIL6-1b*	13	255	450	TC294820	*OsPDIL5-1*	*OsPDIL6-1*	AK063663	119/146 (81.51%)
*TaPDIL7-1a*	37	289	1242	TC287269	*OsPDIL5-2*	*OsPDIL7-1*	AK069367	355/411 (86.37%)
*TaPDIL7-1b*	37	289	1254	TC287269	*OsPDIL5-2*	*OsPDIL7-1*	AK069367	359/416 (86.30%)
*TaPDIL7-1c*	37	289	1242	TC287269	*OsPDIL5-2*	*OsPDIL7-1*	AK069367	356/411 (86.62%)
*TaPDIL7-2a*	53	186	1257	TC287749	*OsPDIL5-3*	*OsPDIL7-2*	ND	311/416 (74.76%)
*TaPDIL7-2b*	53	186	1257	TC287749	*OsPDIL5-3*	*OsPDIL7-2*	ND	311/416 (74.76%)
*TaPDIL8-1*	122	226	1458	TC301351	*OsPDIL5-4*	*OsPDIL8-1*	AK099660	450/485 (92.78%)

^a ^Nomenclature used by Houston *et al*. 2005 [24]; ND = no cDNA sequence available, tBLASTn searches of Gramene identified predicted transcript GRMT00000163510 (OsPDIL5-3/OsPDIL7-2) and the amino acid sequence was predicted from the available genomic sequence Os02g34530 [24].

**Table 2:** Comparison between cDNA sequences from putative homoeologous genes in their coding regions.

Clone pairs	Identity (%)		Substitutions		Indels in frame nt
	nt	aa	nt	aa	
*TaPDIL4-1a/b*	98.73	99.73	14	1	0
*TaPDIL5-1a/b*	97.20	98.41	37	7	0
*TaPDIL6-1a/b*	96.67	96.64	14	5	6
*TaPDIL7-1a/b*	97.90	98.31	24	7	12
*TaPDIL7-1a/c*	99.68	99.52	4	2	0
*TaPDIL7-1b/c*	97.83	98.31	24	7	12
*TaPDIL7-2a/b*	98.17	97.61	23	10	0

### Phylogenetic analysis

The evolutionary relationships between the PDI and PDI-like genes of wheat and of other plants were studied by phylogeny reconstruction based on the alignment of 89 amino acid sequences deduced from the nucleotide sequences of nine genes of wheat, 13 of Arabidopsis, 12 of poplar, 10 of grapevine, 21 of soybean and 12 each of maize and rice. Since the complete genome sequences of Arabidopsis, poplar, grapevine, soybean and rice were available, the dataset used to construct the phylogenetic trees provided the highest confidence of having a representative sample of genes belonging to all the groups of the angiosperm PDI gene family. Moreover, the search in their complete genome sequences retrieved 14 PDI and PDI-like gene sequences in the moss *P. patens *and six genes in the green alga *C. reinhardtii *[48,49]. Since *C. reinhardtii *belongs to the clorophytes, which diverged from the streptophytes (including bryophytes, pteridophytes and angiosperms) over a billion years ago [56], and *P. patens *belongs to the bryophytes, which diverged from the angiosperms about 450 million years ago [57], the inclusion of the sequences from these two species in the phylogenetic analyses allowed to reconstruct a more ancient evolutionary changes in this gene family.

**Figure 1:**
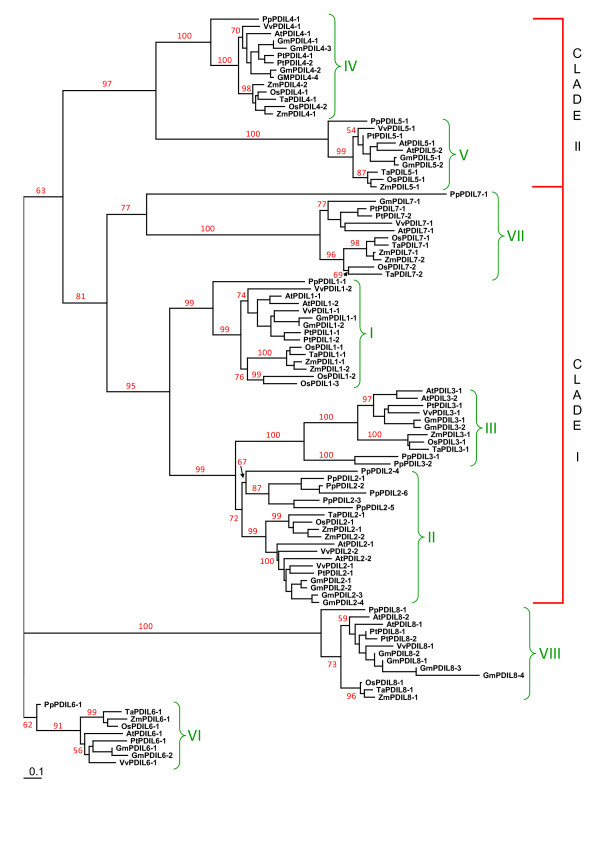
**Phylogenetic tree showing the relationships between the deduced amino acid sequences of PDI and PDI-like genes of different plant species**. The multiple alignment of nine sequences of wheat, 13 of *A. thaliana *(At), 12 of *P. trichocarpa *(Pt), 10 of *V. vinifera *(Vv), 21 of *G. max *(Gm), 12 each of *Z. mais *(Zm) and *O. sativa *(Os) and 14 of *P. patens *(Pp) was performed by ClustalX 1.83 software and the phylogenetic tree was constructed by the neighbour-joining (NJ) method and evaluated by bootstrap analysis (PHYLIP version 3.6). The numbers on the main branches indicate bootstrap percentages for 1,000 replicates. The PDI-like sequences of groups VI and VIII were used as outgroups, due to their high diversification from the other subfamilies. The two major clades (I and II) and the eight phylogenetic groups (I-VIII) indentified in the plant PDI family are highlighted with red and green brackets, respectively.

Four different phylogenetic trees were computed using amino acid sequences of: 1) the whole set of 109 PDI and PDI-like genes of nine species; 2) a set including all the sequences except CrDNJ of *C. reinhardtii*; 3) 103 genes from seven angiosperm species and from *P. patens*; 4) 89 genes from the seven angiosperms. The topology of the four trees was very similar, although the bootstrap values of those obtained with the data sets 2 and 3 were significantly higher than those based on the data sets 1 and 4 (data not shown). The phylogenetic tree shown in Figure 1 was obtained by the alignment including the sequences of the seven angiosperm species and of *P. patens *(data set 3); the nine non-homoeologous PDI and PDI-like sequences of wheat were included into the eight phylogenetic groups (subfamilies) identified in plants, indicating that at least one wheat gene was cloned for each phylogenetic group. Six of the eight subfamilies could be grouped into two major clades, on the basis of the modular structure of the proteins, whereas the subfamilies VI and VIII had highly diversified proteins and thus were considered as outgroups. The first major clade (clade I in Figure 1) included the I (typical PDIs), II and III phylogenetic groups, whose genes encode proteins containing two thioredoxin active domains, located at the N- and C-terminal ends, as well as the VII group, whose members retain only the single N-terminal active domain. For the groups I, II and III the hypothesis of a common evolutionary origin through duplications of a single ancestral gene can be put forward on the basis of their shared structural features, these duplication events would have occurred in the common progenitor of land plants (streptophytes). The close phylogenetic affinity between the genes of subfamily VII, encoding proteins with a single thioredoxin active domain, and those of the subfamilies I, II and III is consistent with the hypothesis that the group VII proteins would have emerged by loss of one of the two thioredoxin active domains of a precursor gene. The second major clade (clade II in Figure 1) comprised the genes of the IV and V phylogenetic groups, whose proteins contain two thioredoxin active domains located in tandem at the N-terminal end. In spite of the global sequence and structural similarities between groups IV and V, the differences at both C-terminal domains and thioredoxin active domains [20] did not allow any inference on their origin and evolutionary relationships. The VI and VIII phylogenetic groups (Figure 1) included genes coding for proteins with a single thioredoxin active domain; on the basis of phylogenetic analysis and domain structure they seem the most divergent genes in the plant PDI family.

Genes from *P. patens*, monocots (wheat, rice and maize) and dicots (Arabidopsis, poplar, grapevine and soybean) formed three distinct sub-clusters within each of the eight PDI phylogenetic groups (Figure 1), indicating that the eight subfamilies would have emerged before the divergence of bryophites and angiosperms. Only three of the six PDI-like genes identified in the genome of *C. reinhardtii *were included in plant phylogenetic groups (CrPDI-4 in group V, CrPDI-5 in group VIII and CrPDI-3 in group VI; Additional file 6), indicating that only three PDI-like genes would be common to both chlorophytes and streptophytes, which diverged over one billion years ago. The phylogenetic trees obtained with the first (not shown) and second data set (Additional file 6) included the protein CrPDI-2 of *C. reinhardtii *in the second major clade, together with proteins of the IV and V phylogenetic groups. On the basis of its domain structure, in particular for the presence of the D or Erp29c domain consisting of a C-terminal α-helical region of about 100 aa, CrPDI-2 was more closely related to the genes of subfamily IV than to those of subfamily V. However, moss and plant genes of the subfamily IV code for proteins containing two thioredoxin active domains that occur in tandem at the N-terminal end, whereas CrPDI-2 lacks a thioredoxin active domain exhibiting an ***a-D ***domain structure. Moreover, the algal protein is about 100 aa shorter than its moss and flowering plant counterparts, a size corresponding to that of a thioredoxin domain. CrRB60 is closely related to the proteins in the II and III phylogenetic groups and is the only *C. reinhardtii *protein included in the first major clade (clade I, Additional file 6). Therefore the four subfamilies of the first major clade (I, II, III and VII) would have been established after the divergence of the streptophytes from the chlorophytes, but before the divergence of the angiosperms from the bryophytes, and would have originated through three duplication events from an ancestral gene similar to those belonging to the II and III phylogenetic groups, followed by the loss of the C-terminal active thioredoxin domain in the protein encoded by one of the four duplicated genes. Apparently genes corresponding to that encoding the protein CrDNJ of *C. reinhardtii *are not present in moss and higher plants. This protein is characterised by the presence of a single active thioredoxin domain and of a N-terminal J-domain, which is characteristic of the proteins belonging to the family Hsp40 of molecular chaperones, whose members regulate the activity of Hsp70s. A blast search showed that proteins with a ***J-a ***domain structure are present only in unicellular green algae, such as *Ostreococcus tauri *and *Micromonas*, and in the protozoa *Paramecium tetraurelia *and *Cryptosporidium hominis*. Also the human protein Erdj5 contains an N-terminal J domain, but it has four active thioredoxin domains [58].

As already mentioned, each of the eight phylogenetic groups included three distinct sub-clusters, each of them containing PDI and PDI-like genes from *P. patens*, monocots and dicots; this would imply that the common ancestor of the streptophytes carried at least eight genes. Moreover, the presence of multiple genes of the same species within single phylogenetic groups can be explained by duplication events occurred either after the separation of the angiosperms from the briophytes or later, after the diversification of monocots and dicots. In fact six of the eight groups (I, IV, V, VI, VII and VIII) included a single sequence of *P. patens*, whereas groups II and III comprised six and two sequences, respectively (Figure 1). The six *P. patens *genes of group II might have been produced by four duplication events, which took place after the divergence from the angiosperms; the most divergent and therefore ancient gene would be *PpPDIL2-4*. Within the same group II the monocot cluster included two genes of maize and a single gene of wheat and rice, indicating that the duplication occurred in maize after its divergence. Soybean was the only dicot species that owned two pairs of similar paralogous genes, most probably derived by two duplications events, whereas Arabidopsis and grapevine had a single pair of paralogous genes and poplar had a single gene.

The monocot genes of group I were represented by three sequences of rice, most probably produced by two duplications, two of maize, deriving from a single duplication, and one of wheat (Figure 1). The sequences of two maize paralogous genes (*ZmPDIL1-1 *and *ZmPDIL1-2*) were very similar, whereas two similar rice sequences (*OsPDIL1-2 *and *OsPDIL1-3*) were very different from the third sequence (*OsPDIL1-1*), suggesting that the first rice duplication would have occurred long before the second one and/or the gene diversification was faster. As for the dicots in group I, Arabidopsis, soybean and poplar had two similar paralogous genes, whereas the two grapevine genes were more diversified. Group IV included a pair of paralogous genes of rice, maize and poplar, whereas in group VIII a single gene duplication was found only in Arabidopsis and poplar. Like in group II, soybean was the only species showing two pairs of paralogous genes in groups IV and VIII. In groups III and V a single gene duplication was found only in soybean and Arabidopsis; finally, soybean showed a pair of close paralogous genes in group VI, whereas all the other sequenced species were represented by a single gene.

It is noteworthy that only group VII hosted two paralogous genes of wheat not related to the allopolyploid origin of its genome; among the monocot of this group, a cluster included *TaPDIL7-1 *of wheat, *OsPDIL7-1 *of rice and two very similar sequences of maize (*ZmPDIL7-1 *and *ZmPDIL7-2*), the second cluster was formed by the genes *TaPDIL7-2 *of wheat and *OsPDIL7-2 *of rice. The presence of at least one gene from both rice and wheat in each of the two minimal monocot clades suggests that a duplication occurred in the common ancestor of these two species, whereas the lack of a maize gene in the clade including *OsPDIL7-2 *and *TaPDIL7-2 *can be explained by a duplication event occurred after maize speciation or by the deletion of the maize gene, although it is not possible to exclude that the maize gene has yet to be cloned. On the other hand, the presence of two maize genes in the first minimal clade may reflect the duplication of the maize genome after its divergence from the other grass species.

The complex patterns of gene duplication and diversification observed within each phylogenetic group could in part be explained by the variable status of whole genome duplication observed in dicots and monocots. Evidence of ancient polyploidization events has been found in virtually all angiosperm genomes investigated [59,60], indicating that all angiosperms may have experienced one or more rounds of genome duplication. On the basis of the complete genome sequence of grapevine, Jaillon et al. [61] have suggested that the common ancestor of grapevine, poplar and Arabidopsis was an ancient hexaploid species that arose after the split between monocots and eudicots. Further genome duplications took place later in Brassicales and in poplar lineages. The recent completion of the soybean genome [62] has brought new evidence to the hexaploid nature of the common ancestor of most eudicot species and has indicated that soybean underwent two additional rounds of whole genome duplication: an allotetraploidisation specific of the soybean lineage, would have occurred approximately 13 million years ago, after a previous duplication which affected the legumes' progenitor about 59 million years ago [63]. Molecular analyses have also indicated a genomic duplication preceding the divergence of the wild relatives of modern cereal grasses, occurred some 60-80 million years ago; during the first part (one-third) of their subsequent evolution limited molecular divergence would have occurred, whereas a marked genomic divergence would have characterized the more recent period (40-55 million years) giving rise to genome size differences ranging from 420 Mb of rice to about 17,000 Mb of wheat [64,65]. It is well established that maize underwent a whole genome duplication event after its divergence from other grasses about 11 millions years ago. Interestingly, also the moss *P. patens *underwent a genome duplication between 30 and 60 million years ago [57,66].

A better understanding of the significance and dynamics of duplication events in the evolution of the PDI gene family in plants can be based on the comparison of the chromosomal locations of PDI and PDI-like sequences in Arabidopsis and poplar. Six of the 13 Arabidopsis sequences, corresponding to the close pairs of paralogous genes included in the I, III and V phylogenetic groups (Figure 1), have been located in duplicated regions of the genome deriving from the most recent polyploidization within the Brassicales. The first event was associated with regions duplicated in chromosome 1 (*AtPDIL1-1 *and *AtPDIL1-2*), the second duplication involved chromosomes 1 and 2 (*AtPDIL5-1 *and *AtPDIL5-2*) and the third one chromosomes 1 and 3 (*AtPDIL3-1 *and *AtPDIL3-2*). The other two pairs of paralogous genes assigned to the II and VIII phylogenetic groups were found on non-duplicated regions of chromosomes 3 and 5 (*AtPDIL2-1 *and *AtPDIL2-2*) and 3 and 4 (*AtPDIL8-1 *and *AtPDIL8-2*); they may have been produced by a more ancient duplication event, which most probably would have occurred after the divergence of the Eurosids I and Eurosid II, which include soybean and Arabidopsis, respectively [67]. In poplar the three closely related pairs of paralogous genes assigned to the I, IV and VIII phylogenetic groups are located in chromosome regions that might represent paralogous segments resulting from the salicoid-specific genome duplication, which occurred about 65 million years ago [68]. *PtPDIL1-1 *and *PtPDIL1-2 *are located in homologous regions of the linkage groups LGII and LGV, *PtPDIL4-1 *and *PtPDIL4-2 *in duplicated regions of LGII and LGXIV, and *PtPDIL8-1 *and *PtPDIL8-2 *in paralogous segments of LGXIII and LGXI. Unfortunately, one of the two closely related paralogous genes of the VII phylogenetic group (*PtPDIL7-2*) was not assigned to any of the 19 linkage groups of the poplar genome. Most probably the three close pairs of paralogous genes observed in maize in our phylogenetic analyses (*ZmPDIL1-1 *and *ZmPDIL1-2*; *ZmPDIL2-1 *and *ZmPDIL2-2*; *ZmPDIL7-1 *and *ZmPDIL7-2*; Figure 1) may have been originated through the whole genome duplication about 11 million years ago, after its divergence from other grasses. The propensity to retain multiple copies of paralogous genes [62,69] may explain the high number of PDI and PDI-like genes found in soybean. Among the analysed species only soybean has two pairs of similar paralogous genes within some of the phylogenetic groups of the plant PDI family (II, IV and VIII), most probably due to the retention of all the four gene copies produced by the two most recent whole genome duplications which affected soybean. According to several studies, the highly duplicated structure of the soybean genome has resulted from an incomplete diploidization due to a low rate of gene evolution and of structural genomic rearrangement [69,70].

### Domain structure of the deduced amino acid sequences of the wheat PDI gene family

**Figure 2:**
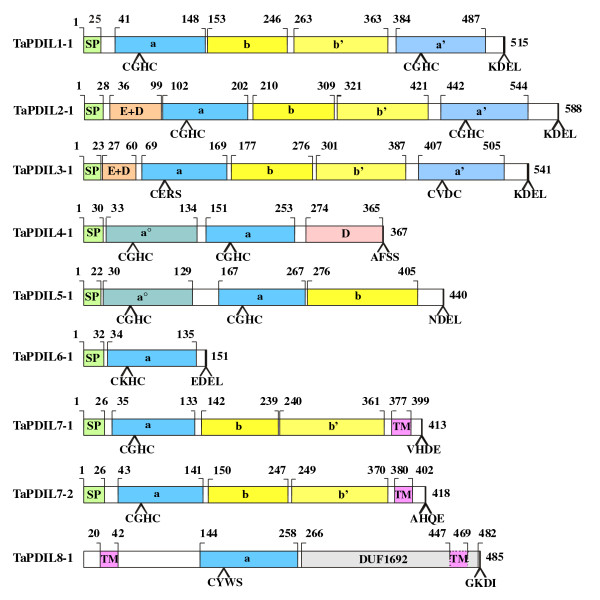
**Domain structure of the deduced amino acid sequences of the wheat PDI and PDI-like genes**. When multiple putative homoeologous cDNA sequences were identified, the domain structure reported in the figure is relative to the cDNA sequence identified by the letter a. The putative signal peptides (SP), the ***a ***and ***b ***type domains, the N-terminal acidic domains (E + D), the ***D ***domains (Erp29c), the transmembrane domains (TM) and the DUF domain of *TaPDIL8-1 *are reported. The thioredoxin-like catalytic site tetra-peptides and the C-terminal tetra peptides are also reported. Numbers above indicate domain boundaries (aa) numbers on the right ORF (aa).

The deduced amino acid sequences of the 14 PDI-like cDNAs isolated in this study (Table 1) and of the three homoeologous cDNAs coding for the typical PDI [34], belonging to nine different homoeologous groups, were searched for conserved motives by comparisons with structurally and functionally characterized sequences in different protein databases (as in Methods). The results are described in Table 3, which reports the structural characteristics of the proteins, whereas Figure 2 shows the domain organization of the wheat proteins of the PDI family encoded by nine genes belonging to different homoeologous groups. The deduced protein sequences of the analysed species encoded by genes clustered into the same phylogenetic group exhibited a high level of structural similarity. The presence of at least a thioredoxin-like domain, which is a feature common to all the PDI and PDI-like proteins, is necessary to accomplish their functional role. The ***a ***and ***a' ***domains are homologous to thioredoxin and contain the -CXXC- catalytic site for isomerase and redox activities, whereas the ***b ***and ***b' ***domains do not show any significant homology to thioredoxin and lack of the -CXXC- active tetrapeptide; nevertheless the secondary structure of all four domains is similar to that of the thioredoxin. The surface-exposed N-terminal cysteine of the -CXXC- active site tetrapeptide is responsible for the direct interaction with the substrate and is needed for any thiol-disuphide reaction, whereas the C-terminal cysteine of the tetrapeptide is required for many thiol-disuphide exchange reactions to proceed efficiently and makes mixed disulphide intermediates with substrate very transient [5]. Moreover, for thioredoxin modifications in the intervening amino acids affect the redox potential and determine the oxidant or reducing properties of the enzyme [71,72]. The most common active-site motif -CXHC-, typically -CGHC-, is found in efficient thiol-disulphide oxidants of the ER and bacterial periplasm [5]. Besides the active site tetrapeptide, there are three additional prominent determinants of the PDI-family members enzymatic activity: 1) the presence/absence of additional residues modulating the pK_a _of the active-site cysteines; 2) the presence/absence of a glutamic acid-lysine charged pair that is involved in proton transfer reaction; 3) a high-affinity substrate-binding site in a non catalytic domain that is essential for isomerization reactions [5]. For instance, a conserved arginine that is present in many members of the PDI family has been reported to modulate the pK_a _of the active-site cysteine residues by moving into and out of the active-site locale [73]. This motion is involved in the timing mechanism that allows a single catalyst to act as an efficient isomerase and oxidase of protein substrates and facilitate the release of non-productive folding substrates. This arginine is important for the catalysis of oxidation by PDI, ERp57, ERp72 and P5 and is also conserved in most of the other human PDI-family member ***a***-like domains [5]. In addition to a -CXXC- active site and a modulation of the pK_a _values of the active-site cysteines, efficient completion of the catalytic cycle for oxidation or reduction requires numerous proton transfer reactions both within the catalyst and to and from the substrate [5]. In the thioredoxins, a buried, charged glutamic acid-lysine pair that is located under the CXXC active site is important for the catalytic activity of thioredoxin [74] and for the oxidative activity of PDI and ERp57 [5]. Finally, detailed *in vitro *enzymology on linear combinations of PDI domains has shown that the isolated ***a ***and ***a' ***domains can catalyse thiol-disulphide exchange reactions in peptide and protein substrates, whereas a combination of a catalytic domain and of the ***b' ***domain is required for simple isomerization reactions. In fact the ***b' ***domain contains a high affinity binding site by which PDI holds the substrate during isomerisation reactions. On the contrary, since the ***b ***domain of human PDI has never been implicated in substrate binding, its meaning seems mostly structural, without a direct catalytic role [75]; moreover, its addition does not influence the catalytic ability of the PDI domain constructs [76].

**Table 3:** Characteristics of the wheat PDI-like proteins.

Name	Length	Mw	pI	N-glycisilation sites (putative)	Domain composition^a^	Active site sequence	Conserved charge pair sequence^b^	Conserved arginine^b^
TaPDIL1-1a	515	56.59	4.99	1: N283	a-b-b'-a'	CGHC, CGHC	E62-K96, E406-K439	R136, R475
TaPDIL1-1b	512	56.44	5.03	1: N283	a-b-b'-a'	CGHC, CGHC	E62-K96, E406-K439	R136, R475
TaPDIL1-1c	515	56.63	4.96	1: N283	a-b-b'-a'	CGHC, CGHC	E62-K96, E406-K439	R136, R475
TaPDIL2-1	588	63.80	4.61	2: N109, N212	c-a-b-b'-a'	CGHC, CGHC	E123-K157, E464-K497	R193, R535
TaPDIL3-1	541	59.61	4.95	1: N150	c-a-b-b'-a'	CERS, CVDC	*L90*-K124, E429-R462	R160, O492
TaPDIL4-1a	367	40.27	6.17	0	a°-a-D	CGHC, CGHC	E54-K87, E173-K211	R125, R244
TaPDIL4-1b	367	40.26	6.17	0	a°-a-D	CGHC, CGHC	E54-K87, E173-K211	R125, R244
TaPDIL5-1a	440	47.15	5.12	1: N170	a°-a-b	CGHC, CGHC	E51-K89, E188-K226	R119, R257
TaPDIL5-1b	440	47.22	5.36	1: N170	a°-a-b	CGHC, CGHC	E51-K89, E188-K226	R119, R257
TaPDIL6-1a	151	16.99	4.96	0	a	CKHC	*Q56-S95*	R126
TaPDIL6-1b	149	16.65	5.30	0	a	CKHC	*Q54-S93*	R124
TaPDIL7-1a	413	46.30	4.91	1: N275	a-b-b'-t	CGHC	D56-K90	R126
TaPDIL7-1b	417	46.62	4.91	2: N176, N279	a-b-b'-t	CGHC	D60-K94	R130
TaPDIL7-1c	413	46.32	4.87	2: N172, N275	a-b-b'-t	CGHC	D56-K90	R126
TaPDIL7-2a	418	46.40	5.12	0	a-b-b'-t	CGHC	D64-K98	R134
TaPDIL7-2b	418	46.34	5.03	1: N384	a-b-b'-t	CGHC	D64-K98	R134
TaPDIL8-1	485	54.41	6.90	ND	t-a-t	CYWS	N164-K203	R249

^a ^a = active site containing thioredoxin-like domain; b = inactive thioredoxin-like domain (note that the superscript are included to distinguish between domains of proteins containing more than one a and b domain on the basis of their position and not on the basis of sequence homology); c = acidic segment; D = Erp29c domain; t = transmembrane domain. ^b ^The position of conserved charge pair sequence and arginine residues that are considered important for the catalytic activity of different members of the human PDI family are determined on the basis of multiple alignments of the ***a ***type domains of wheat PDI-like proteins and human classical PDI [Accession number P07237] (Figure 3). ND = not determined because TaPDIL8-1 lacks a putative N-terminal signal peptide. Proteins without signal peptides are unlikely to be exposed to N-glycosilation machinery and thus may not be glycosylated in vivo even though they contain potential motifs.

**Figure 3:**
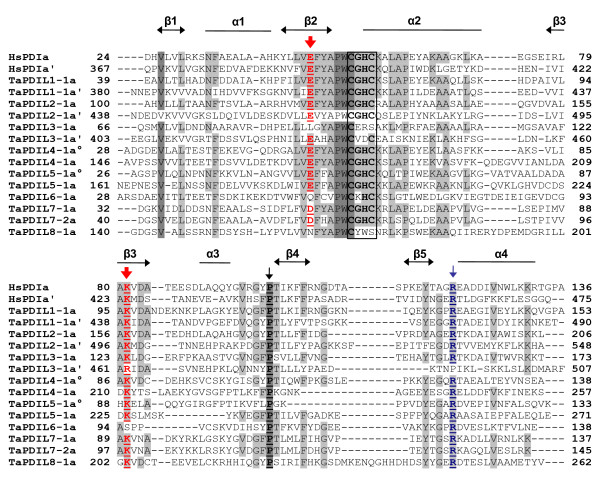
**Multiple sequence alignment of the *a*-type domains of wheat PDI-like proteins and human typical PDI**. The alignment was constructed using ClustalX version 1.83 (Thompson et al. 1997) with the following parameters: gap penalty 10, gap extension penalty 0.5, BLOSUM protein weight matrix. The alignment was also manually adjusted, taking the secondary structure predictions of wheat PDI-like proteins and the known structure of the ***a ***domain of the human PDI into account. Residues highlighted in grey and light grey showed 100% and > 50% identity conservation, respectively. The elements of the secondary structure are specified by open bars (α helices) and arrowheads (β strands). Red arrows indicate the two buried charged residues in the vicinity of the active site, a blue arrow indicates the conserved arginine (R) located in the loop between β5 and α4 of the catalitic domains and a black arrow the cis prolines (P) near each active site. Active-site residues within the ***a ***type domains are boxed.

The typical PDI proteins encoded by the three homoeologous genes (TaPDIL1-1a/c) had a multidomain structure comprising four sequential thioredoxin-like domains (***a-b-b'-a'***); moreover they had both the N-terminal signal peptide and the C-terminal KDEL signal, which are responsible respectively for their translocation and retention into the endoplasmic reticulum (ER) (Table 3 and Figure 2). Both the ***a ***and ***a' ***domains contained the -CGHC- motif of the active site and the conserved arginine residues (R136 and R475) (Table 3 and Figure 3), which are involved in the regulation of the active site redox potential in human PDI [5,73]. In addition, two charged glutamic acid-lysine pairs (E62-K96 and E406-K439) were also conserved; they were located close to each -CGHC- active site and, as mentioned before, are important for the catalytic activity of the thioredoxin [74] and for the oxidative activity of the human PDI [5].

TaPDIL2-1 and TaPDIL3-1 had a multidomain organization similar to that of the typical PDI, with the addition of a N-terminal domain named ***c***, which is rich of acidic residues (Figure 2); thus the domain distribution was:***c*-*a-b-b'-a'***. Remarkably, the ***c ***domain was present in all the members of the II and III phylogenetic groups and was reminiscent of the ***c ***domain found close to the C-terminus of the typical PDIs of mammals and to the N-terminus of homologs of ERp72 [5,19]. In mammals the ***c ***domain is a putative low affinity and high capacity calcium-binding site; consequently, the PDI-like proteins of the II and III phylogenetic groups could participate to calcium storage within the cell or, alternatively, interact with other proteins or peptides at this specific domain. RB60 of *C. reinhardtii *was a PDI-like protein with a ***c*-*a-b-b'-a' ***domain structure similar to that of the plant members in the II and III phylogenetic groups and was located in the chloroplasts and in the ER. Levitan et al. [30] have demonstrated that a 50 aa long leader sequence of RB60 is responsible for its correct targeting to chloroplast and ER and is cleaved upon its translocation into the ER, whereas it is retained after its import to the chloroplasts, where RB60 appears associated with the thylakoid membranes; this association likely depends from the acidic domain in the N-terminus of the protein. TaPDIL2-1 and TaPDIL3-1 were the largest (588 and 541 aa, respectively) PDI-like proteins identified in wheat (Table 3) and are predicted to be secretory proteins, being characterised by the presence of a putative N-terminal signal peptide and of the C-terminal KDEL signal for ER retention (Figure 2). Like their orthologous proteins of wheat, the soybean PDI proteins of the I, II and III phylogenetic groups (GmPDIL-1, GmPDIL-2, GmPDIL-3a and GmPDIL-3b) contained an N-terminal signal sequence for their targeting to the ER and a C-terminal ER retention sequence KDEL; in fact they have been located in the soybean ER lumen [26,28]. Both the ***a ***and ***a' ***domain of TaPDIL2-1 contained the -CGHC- common active site motif, moreover the arginine residues (R193 and R535) and the glutamic acid-lysine pairs (E123-K157 and E464-K497) were conserved (Table 3 and Figure 3). Instead, TaPDIL3-1 showed two non-characteristic tetra-peptide active sites (CERS and CVDC), probably affecting its redox potential and consequently its function. In addition, the glutamic acid in the ***a ***domain, the presumed proton acceptor of the charged pair near the -CERS- active site, was replaced by a leucine residue, whereas in the ***a' ***domain the arginine, which is critical for the catalytic function of the human PDI, was replaced by a proline residue (Table 3 and Figure 3).

The PDI-like proteins encoded by putative homoeologous members of the two paralogous genes (*TaPDIL7-1 *and *TaPDIL7-2*) belonging to the VII phylogenetic group (TaPDIL7-1a, TaPDIL7-1b and TaPDIL7-1c; TaPDIL7-2a and TaPDIL7-2b) showed the same multidomain organization of the typical PDI (TaPDIL1-1), but they lacked the ***a' ***C-terminal domain, as well as the KDEL signal (Figure 2). Moreover, the wheat PDI-like proteins of group VII were characterised by the presence of a C-terminal transmembrane segment, which could retain the protein in the ER through its anchoring to the membrane, even in the absence of the KDEL signal. The N-terminal thioredoxin active domain of both group VII proteins showed the three prominent determinants of the enzymatic activity of the PDI family members: the most common active site -CGHC-, the conserved arginine residue and the charged pair near the active site; the only exception was represented by the replacement of the glutamic acid with an aspartic acid residue, even though they have similar chemical properties (both are negatively charged and hydrophilic) (Table 3 and Figure 3). Transmembrane proteins with the ER-luminal region characterised by an ***a-b-b' ***multidomain organization have also been identified in man, *Drosophila *and *Caenorhabditis elegans *[19,77].

The wheat PDI-like proteins encoded by the two putative homoeologous genes of the IV phylogenetic group (TaPDIL4-1a and TaPDIL4-1b) possessed a putative N-terminal secretory signal sequence and two tandem thioredoxin domains, enclosing a -CGHC- active site (Figure 2 and Table 3); moreover they had conserved arginine residues (R125 and R244) and charged glutamic acid-lysine pairs (E54-K87 and E173-K211), which are important for their enzymatic activity (Table 3 and Figure 3). TaPDIL4-1a and TaPDIL4-1b, like all plant PDI-like proteins in the IV phylogenetic group, had an additional α-helical domain of about 100 aa, termed D domain, whose function is unknown. Proteins with ***a°-a-D ***modular structure have been identified in the amoeba *Dictyostelium discoideum *[78] and in the fungi *Aspergillus niger *and *Neurospora crassa *[79], but not in mammals, where proteins with a C-terminal D domain, like the human ERp28 or rat ERp29, are redox inactive, being characterised by a ***b-D ***modular structure [19]. As mentioned before, the PDI-like protein CrPDI-2 of *C. reinhardtii *contained the D domain, but lacked a thioredoxin active domain exhibiting an ***a-D ***domain structure. In spite of the presence of a potential ER-translocation signal, the plant PDI-like proteins of the IV phylogenetic group differed from those isolated in fungi for the absence of an ER-retention signal, suggesting that they might be targeted to a different subcellular location or be retained, as part of a heteromeric complex, with other subunits containing such a signal. However, in *D. discoideum *it has been shown that the C-terminal part of the ***D ***domain is responsible for the ER retention of the PDI-D protein [80], suggesting that the plant PDI-like proteins of the IV phylogenetic group, all containing a ***D ***domain, may be retained in the ER. By confocal microscopy it has been shown that the two soybean PDI proteins belonging to the IV phylogenetic group (GmPDIS-1 and GmPDIS-2) are located in the ER of cotyledon cells [25]; they have also been detected in protein storage vacuoles (PSVs) of mature seed cotyledons, although how these PDI proteins are transported from the ER to the PSV and what role they play in the PSV is yet unknown [25].

TaPDIL5-1a and TaPDIL5-1b possessed two tandem thioredoxin active domains (***a°-a***), each containing the typical tetra-peptide site -CGHC-, the conserved arginine residues (R119 and R257) and the charged glutamic acid-lysine pairs (E51-K89 and E188-K226) near the active site, and an inactive thioredoxin ***b ***domain at their C-terminus (Table 3 and Figure 3). Moreover, they contain the signal peptide and a modified NDEL signal for retention in the ER [81] (Figure 2). The soybean PDI protein of the V phylogenetic group (GmPDIM) has been shown to be an ER luminal protein, it possesses a putative signal peptide and the classical C-terminal ER-retention signal sequence KDEL [27]. All the deduced amino acid sequences of the proteins of the V phylogenetic group were tightly related to the mammalian P5 PDI-like proteins [19] in terms of sequence homology (about 40% identity), multidomain organization and length.

TaPDIL6-1a and TaPDIL6-1b were encoded by two putative homoeologous genes of the VI phylogenetic group and were the smallest PDI-like proteins identified in wheat, their size corresponding to that of the other plant PDI-like sequences of the VI group (146-150 aa). Their modular structure was very simple, being characterised by the presence of a single thioredoxin domain containing a non-common tetra-peptide active site (CKHC) (Figure 2). Furthermore, the charged glutamic acid-lysine pair near the active site was replaced by a pair of uncharged polar amino acids, such as glutamine and serine (Q56-S95) (Table 3 and Figure 3). The mutated tetrapeptide active site and the absence nearby of the charged glutamic acid-lysine pair involved in proton transfer reactions were peculiar to all the plant PDI-like proteins of the VI group and probably affect their redox potential and consequently their function. TaPDIL6-1a and TaPDIL6-1b contained the signal peptide and a modified EDEL signal for retention in the ER [82]. Interestingly, some plant PDI-like proteins of the VI phylogenetic group, such as OsPDIL6-1 and ZmPDIL6-1, lack a KDEL-like signal, being characterised by the presence, respectively, of the C-terminal tetrapeptides LQDS and LEAD, for which there is no evidence of their involvement in ER retention. Consequently, these proteins may be either located in a different cell compartment or retained within the ER through an unknown mechanism. In maize mutants such as *floury-2 *(*fl2*), *mucronate *(*Mu*) and *defective endosperm B30 *(de*-B30), which are affected by ER stress due to the wrong processing of seed storage proteins (α- e γ-zeins), the PDI-like protein encoded by the single maize gene *ZmPDIL6-1 *of the group VI showed very high expression, similar to that of typical PDI (ZmPDIL1-1), BiP and calnexin [20]. However, the behaviour of the protein encoded by the gene *ZmPDIL6-1 *is different from that of other chaperons, because it is located neither within the luminal portion of the ER, nor in the protein bodies. Further studies will be needed to elucidate whether the location within the ER of PDI-like proteins of the VI group is related to the accomplishment of any specific function, or it is just transient and then they are moved to other cell compartments.

Also TaPDIL8-1 contained a single thioredoxin active domain with a mutated tetrapeptide sequence (CYWS) (Figure 2 and Table 3), moreover the glutamic acid, the presumed proton acceptor of the charged pair near the active site, was replaced by an asparagine residue (Table 3 and Figure 3). Like all the plant PDI-like proteins of the VIII phylogenetic group, TaPDIL8-1 lacked both the N-terminal signal peptide and the C-terminal KDEL signal, but it carried two transmembrane regions, one in the N-terminus and one in the C-terminus. Moreover, it had a C-terminal DUF1692 domain, which is present in several proteins of unknown function; three of them (Erv41p and Erv46p of yeast and ERGIC-32 of mouse) have partially been characterised and are involved in the protein transport between ER and Golgi complex. Yeast Erv41p and Erv46p are selectively and efficiently packed into COOPII vesicle and cycled between ER and Golgi [83]. Like ERGIC-32 of mouse, the yeast proteins lack a signal peptide sequence and are predicted to be transmembrane proteins, with large luminal and very short N- and C-terminal cytosolic domains. However, so far it is unknown the precise molecular mechanism by which these proteins regulate the membrane trafficking between ER and Golgi. ERGIC-32 has been located in the ER-Golgi intermediate compartment (ERGIC) [84], a complex of tubulovescicular membrane system between the rough ER and the Golgi, which in mammals is the first anterograde/retrograde sorting station in the secretory pathway [85]. Several lines of evidence suggest that protein sorting is only one of the ERGIC functions, but other presumed roles may be present, although less evident. The ERGIC appears involved in the quality control of newly synthesized proteins, as indicated by the presence of the KDEL receptor, which cycles chaperones and folding enzymes escaped from the ER back to this compartment; moreover, it would be involved in the retrotranslocation to the cytosol of permanently misfolded proteins. A role of the ERGIC has also been postulated in the folding of the newly ER synthesized proteins before their translocation to the Golgi complex. Further studies will be necessary to understand whether the plant PDI-like proteins belonging to the VIII phylogenetic group are localized in a compartment similar to the ERGIC of mammals and to investigate their function.

The functional features of the proteins encoded by the PDI gene family of wheat can be predicted on the basis of their domain structure and on the presence/absence of the four major determinants whose role has been identified, as previously described, in humans. The presence of a -CXHC- active site in combination with the three other determinants of enzymatic activity suggest that TaPDIL1-1, TaPDIL2-1 TaPDIL7-1 and TaPDIL7-2 would be involved in disulphide bond formation. Since both TaPDIL4-1 and TaPDIL5-1 lacked the ***b'***-like domain, but retained the other features and the -CXHC- active site, they would be expected to be efficient oxidases. Due to the presence of a ***D ***domain, TaPDIL4-1 should also possess isomerase activity. In fact it has been shown that the ***D ***domain of Wind of Drosophila, encoded by a gene ortholog of *ERp29 *of rat, contains a binding site for its substrate [86]. These predictions are in line with recent studies on the oxidative refolding activity of the recombinant PDI proteins of soybean GmPDIL-1, GmPDIL-2, GmPDIS-1/2 and GmPDIM, which are orthologous to the wheat proteins TaPDIL1-1, TaPDIL2-1, TaPDIL4-1 and TaPDIL5-1. The five soybean proteins are able to catalyze the oxidative refolding of reduced and denatured Rnase A, although with different efficiency: the activity of GmPDIM, GmPDIS-1 and GmPDIS-2 was approximately 10% of GmPDIL-1 and 20% of GmPDIL-2 [25-27]. These data indicate that the domain architecture of the PDI proteins and most probably the presence of the ***b' ***domain plays an important role in their oxidase and isomerase activities, at least *in vitro*. The five soybean PDI proteins have oxidative folding activities *in vitro *and are located in the cotyledon ER, consequently they could be involved in the folding of seed storage proteins such as glycinin and β-conglycin. Moreover, four of them, except GmPDIM, function as molecular chaperones, by preventing the aggregation of amyloid β-peptide (Aβ) (1-40) monomers (GmPDIS-1/2) or unfolded rhodanase (GmPDIL-1 and GmPDIL-2) [25,26]. Data from coimmunoprecipitation and crosslinking experiments have shown that the five PDI soybean proteins may play different roles in the folding of storage proteins. Under normal conditions an association has been detected between GmPDIS-1 and GmPDIM and either proglycinin or β-conglycin [25,27], indicating their primary function in the folding of the storage proteins. However, a strong association of GmPDIL-1 and GmPDIL-2 with the proglycinin and the β-conglycin α-subunit was detected in the cotyledon cells under conditions that disrupt the folding of the proteins in the ER, such as the presence of dithiothreitol or tunicamycin [26]; this suggests the role of GmPDIL-1 and GmPDIL-2 as molecular chaperones, involved in the folding or quality control of the seed storage proteins.

TaPDIL6-1 possessed both the -CXHC- active site and the conserved arginine residue; however the glutamic-acid proton acceptor was absent, suggesting that its activity as potential catalyst of oxidation reactions would be relatively inefficient. In fact, *in vitro *analysis of ERp18, a human PDI-like protein whose structural features are similar to TaPDIL6-1, showed that its oxidase activity was only 15% of the ***a ***domain of the human PDI [82]. Both the N-terminal ***a ***domains of TaPDIL3-1 and TaPDIL8-1 lacked the conserved glutamic acid proton acceptor and the C-terminal cysteine in their active sites. Due to the unusual structure and catalytic motif of TaPDIL8-1 it is difficult to predict its functional role, whereas it is possible to hypothesize that TaPDIL3-1 could retain at least partially its original function. Accordingly, the N-terminal ***a' ***domain of TaPDIL3-1, apart the unusual intervening amino acid at the active site sequence, retained the other determinants of enzymatic activity. However, the soybean PDI proteins GmPDIL-3a and GmPDIL-3b, orthologous to TaPDIL3-1, do not show any oxidase or reductase activitiy *in vitro *[28]. Replacement of the second and third amino acid in standard redox active -CGHC- motifs found in the ***a' ***domain of GmPDIL-3a and GmPDIL-3b and in all the plant PDI proteins of the III phylogenetic group may explain the lack of such enzymatic activities. Alternatively, the absence of other amino acids such as arginine, which is important for the regulation of the redox potential of the active site in human PDI, may cause the lack of enzymatic activity. In addition, neither GmPDIL-3a nor GmPDIL-3b showed chaperone activity *in vitro *and interaction between these PDI proteins and storage proteins (such as proglycinin and β-conglycin) and other ER molecular chaperones (such as calnexin, calreticulin, BIP and PDI family proteins) was not detected *in vivo *[28], suggesting that GmPDIL-3a and GmPDIL-3b may not act as chaperones in the ER. However, a fraction of GmPDIL-3a and GmPDIL-3b formed complexes with unidentified proteins in the ER in both thiol-dependent and thiol-independent ways. Identification of partner proteins in the mixed disulfide and non covalent complexes of GmPDIL-3a and GmPDIL3-b would be important to clarify the biochemical and physiological functions of plant PDI proteins belonging to the III phylogenetic group.

### Chromosome location of the wheat PDI-like genes and syntenic relationships with their orthologous genes of rice

**Figure 4:**
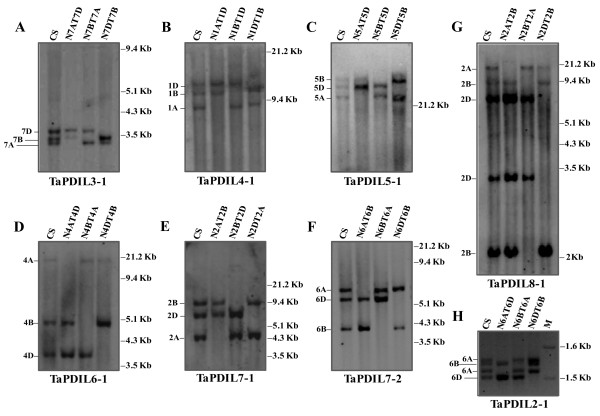
**Chromosome location of wheat PDI-like genes**. (A-G) Southern analysis of DNA of CS and its NT lines of homoeologous groups 7 (A), 1 (B), 5 (C), 4 (D), 2 (E and G) and 6 (F) digested with *Nco*I (A), *Sac*I (B), *Eco*RV (C), *Hin*dIII (D and G), *Eco*RI (E) and *Dra*I (F) and hybridised with specific probes of seven PDI-like sequences: TaPDIL3-1 (A), TaPDIL4-1 (B), TaPDIL5-1 (C), TaPDIL6-1 (D), TaPDIL7-1 (E), TaPDIL7-2 (F) and TaPDIL8-1 (G). The deduced chomosome location is indicated on the left side. The sizes of the molecular-weight marker (kb) are shown on the right side. (H) Gel electrophoresis in a 2% agarose gel of PCR products amplified from DNA of CS and its NT lines of homoeologous group 6 with a specific primer pair designed at the 3' region of the TaPDIL2-1sequence. The deduced chromosome location is indicated on the left side. Size of the molecular-weight marker bands (kb) are shown on the right side.

The wheat gene sequences encoding the typical PDI (TaPDL1-1) had previously been located by aneuploid analysis in chromosome arms 4AL, 4BS and 4DS of bread wheat [34]. The chromosome location of genes corresponding to the eight novel full-length PDI-like cDNA sequences isolated in this study were determined through Southern analysis of DNA from CS and its nulli-tetrasomic (NT) lines. For each PDI-like sequence the optimal combination of restriction enzyme and gene specific probe was previously selected on the basis of Southern analysis of CS DNA (data not shown) and then used for aneuploid analysis with the NT lines of CS. Except for *TaPDIL2-1*, the Southern patterns of seven PDI-like sequences allowed the chromosome assignment of all their hybridization fragments and showed that the corresponding genes were present in single copy for each of the three genomes (Fig 4A-G). The following combinations of restriction enzymes and gene specific probes were used in Southern analysis of CS and its aneuploid lines: 1) *Nco*I, *TaPDIL3-1*; 2) *Sac*I, *TaPDIL4-1*; 3) *Eco*RV, *TaPDIL5-1*; 4) *Hin*dIII, *TaPDIL6-1*; 5) *Eco*RI, *TaPDIL7-1*; 6) *Dra*I, *TaPDIL7-2*; all the hybridisation patterns consisted of three fragments corresponding to the three homoeologous genes of the allohexaploid wheat genome (Figure 4A-F). On the basis of NT analysis the three hybridisation fragments were located in the following chromosome homoeologous groups: 1) *TaPDIL3-1*, group 7; 2) *TaPDIL4-1*, group 1; 3) *TaPDIL5-1*, group 5; 4) *TaPDIL6-1*, group 4; 5) *TaPDIL7-1*, group 2; 6) *TaPDIL7-2*, group 6 (Figure 4A-F). For *TaPDIL8-1 *the simplest hybridisation pattern resulted from the digestion of CS DNA with *Hin*dIII and consisted of five fragments, one located in chromosome 2A, two in 2B and two in 2D (Fig 4G). This complex hybridisation pattern was caused by an internal *Hin*dIII restriction site in the homoeologous sequences of *TaPDIL8-1 *located in chromosomes 2B and 2D, as confirmed also by restriction analysis of the cloned genomic sequence, corresponding to one of the three homoeologous genes.

For *TaPDIL2-1 *no simple restriction pattern could be identified with all enzyme/gene specific probe combinations tested, but it was evident that only the chromosomes of homoeologous group 6 were involved. To confirm this assignment, the genomic DNA of CS was amplified by primer pairs designed in different regions of the sequence; several different amplification patterns displaying from one to four fragments were obtained by electrophoresis in 2% agarose gel (data not shown). The primer pair flanking a region spanning the last 3 introns of *TaPDIL2-1*, which displayed the best pattern consisting of four fragments of about 1,5 kb, was subsequently used to amplify the NT lines of CS. Aneuploid analysis located the four amplification products in the chromosomes of the homoeologous group 6 (Figure 4H), with the smallest amplicon located in chromosome 6D, the second largest in 6B and the remaining two in 6A, showing that a duplication event involving *TaPDIL2-1 *took place in the genome A.

Considering the main syntenic relationships between rice and wheat chromosomes, the locations of the eight PDI-like genes of wheat are compatible with those of their orthologous genes of rice, as shown in Additional file 7. The seven wheat homoeologous groups (w1-w7) were in fact syntenic to the 12 rice chromosomes (r1-r12), in particular w1 = r5 + r10, w2 = r7 + r4, w3 = r1, w4 = r3 + r11, w5 = r12 + r9 + r3, w6 = r2 and w7 = r6 + r8 [87]. Since the order of single copy rice genes is remarkably conserved across the wheat bins [88], in spite of the extensive duplications and rearrangements occurred in both species [89], it was decided to exploit the outputs of the sequence-based macrocolinearity between the MSU Rice Pseudomolecules (Release 6.1) [90] and Wheat EST markers, whose location on the wheat Bin map is reported in GrainGenes [91], to asses the syntenic relationships between the regions of the wheat and rice chromosomes flanking the PDI-like genes. The search of the flanking Wheat Bin Mapped Markers [92] reported onto Rice Pseudomolecules [93], for each orthologous PDI-like gene of rice [20], indicated for six of them (*TaPDIL3-1*, *TaPDIL4-1*, *TaPDIL5-1*, *TaPDIL7-1*, *TaPDIL7-2 *and *TaPDIL8-1*), the presence of several flanking markers in the regions surrounding the orthologous genes of rice; they were located in the same homoeologous group wherein the wheat PDI-like genes had previously been assigned through Southern analysis (Additional file 7). In two cases, the identified marker of rice coincided with the searched gene: *TaPDIL5-1*/CSU223BE399897 and *TaPDIL7-2*/UCD078BF201426. The rice gene *OsPDIL2-1 *(*OsPDIL1-4 *in [20]), orthologous to *TaPDIL2-1 *of wheat, was located at the extreme end of rice chromosome 2, where no Wheat Bin Mapped Marker was found. The closest available markers were reported on the rice Pseudomolecule further downstream of *OsPDIL2-1*; nevertheless all of them mapped to the short arm of the group 6 homoeologous chromosomes of wheat, the same location of *TaPDIL2-1 *determined through Southern and PCR analyses (Additional file 7). Only for the rice gene *OsPDIL6-1 *(also named *OsPDIL5-1*), whose orthologous wheat gene *TaPDIL6-1 *was located in group 4 homoeologous chromosomes, the closest identified marker KSU035BE406977 mapped to the group 1 homoeologous chromosomes; nevertheless, all the other identified flanking markers mapped into the group 4 chromosomes. Besides confirming the chromosome locations previously identified by NT lines, the analysis permitted the assignment of the PDI-like genes to specific arms of the wheat homoeologous chromosomes. The chromosome arm location and homoeologous groups were as follows: *TaPDIL2-1*: 6S; *TaPDIL3-1*: 7S; *TaPDIL4-1*: 1S; *TaPDIL5-1*: 5L; *TaPDIL7-1*: 2L; *TaPDIL7-2*: 6L; *TaPDIL8-1*: 2S. *TaPDIL6-1 *was located in the short arm of chromosome 4A and in the long arms of chromosomes 4B and 4D; this apparent anomaly can be explained by a well known pericentric inversion occurred in wheat chromosome 4A [94]. In conclusion present study confirmed the high level of synteny between rice and wheat chromosomes, opening opportunities for saturation of specific chromosome regions of wheat exploiting rice markers and subsequent chromosome walking and gene positional cloning.

### Genomic organization of the wheat PDI-like genes

The genomic sequences encoding the eight novel PDI-like genes were amplified by the same primer combinations which had previously been used to clone the corresponding cDNA sequences. Two independent PCR reactions were performed for each primer combination, the products of both amplifications had the same electrophoretic mobility. Four cloned amplicons for each PDI-like gene were partially sequenced at their ends using universal primers; a single clone for each gene was chosen and both strands were completely sequenced using internal primers. Therefore, for each of the eight novel PDI genes only one genomic clone, corresponding to one of the homoeologous genes, was completely sequenced and its structure was compared with that of other plant PDI genes.

**Figure 5:**
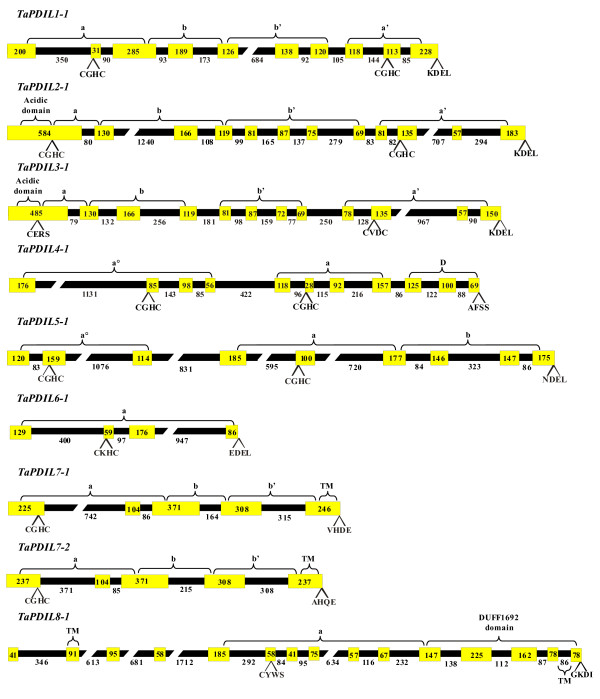
**Schematic representation of the intron-exon structures of the wheat PDI and PDI-like genes**. Only the ORF sequences have been reported, whereas the 5' and 3' UTRs have been omitted. Exons are indicated by yellow boxes, introns by black boxes, numbers represent their length (bp). Tetrapeptide sequence and position of the thioredoxin-like catalytic sites are indicated as well as the C-terminal tetrapeptide. Exons encoding each identified domain are labelled by braces.

The lengths of the genomic sequences of the eight non-homeologous PDI-like genes were: 1) *TaPDIL2-1*: 5213 bp; 2) *TaPDIL3-1*: 4294 bp; 3) *TaPDIL4-1*: 3820 bp, 4) *TaPDIL5-1*: 5326 bp 5) *TaPDIL6-1*: 2162 bp; 6) *TaPDIL7-1: *2887 bp; 7) *TaPDIL7-2*: 2475 bp; 8) *TaPDIL8-1*: 7034 bp; they were aligned with the corresponding cDNA sequences to determine the exon-intron structure. Sequence alignment showed an almost perfect nucleotide match between the cDNA sequences and the corresponding exonic regions of the genomic sequences and evidenced the complex genomic organization of the eight cloned PDI-like genes of wheat. Their genomic structure included the following exon numbers: 12 in *TaPDIL2-1 *and *TaPDIL3-1*; 11 in *TaPDIL4-1*; 9 in *TaPDIL5-1*; 4 in *TaPDIL6-1*; 5 in *TaPDIL7-1 *and *TaPDIL7-2*; 15 in *TaPDIL8-1 *(Figure 5). The comparison between the exon/intron structure and the domain architecture of the wheat PDI and PDI-like genes revealed that the ends of some domains corresponded to the exon/intron junctions (***a' ***in *TaPDIL1-1, TaPDIL2-1 *and *TaPDIL3-1*; ***a° ***in *TaPDIL4-1 *and *TaPDIL5-1*; ***a ***in *TaPDIL4-1*), whereas some other domain boundaries fell within the same exon (***a***, ***b ***and ***b' ***in *TaPDIL1-1*, *TaPDIL2-1, TaPDIL7-1*, *TaPDIL7-2*; ***a ***and ***b ***in *TaPDIL3-1 *and *TaPDIL5-1*) (Figure 5); the ***D ***domain in *TaPDIL4-1 *and the two transmembrane domain in *TaPDIL8-1 *were structurally independent. When present the signal peptide and the acidic domain were always located in the first exon and structurally linked to the most N-terminal ***a ***domain.

The PDI and PDI-like genes of wheat, Arabidopsis, rice and *P. patens *belonging to the same phylogenetic group revealed a high level of conservation of their structural features, in terms of intron pattern and exon number, size and position of the active sites (Additional file 8); consistent with previous studies [95,96]; the corresponding genes of the alga *C. reinhardtii *showed a very different intron-exon structure (data not shown).

As reported in the phylogenetic analysis section, the first phylogenetic group (typical PDI) included a single gene of wheat (*TaPDIL1-1*), two of Arabidopsis (*AtPDIL1-1 *and *AtPDIL1-2*) and three of rice (*OsPDIL1-1*, *OsPDIL1-2 *and *OsPDIL1-3*), four of them (*TaPDIL1-1*, *AtPDIL1-2*, *OsPDIL1-1 *and *OsPDIL1-2*) consisting of ten exons. Since this same gene structure is present in the three species, it is plausible that the progenitor species of Monocots and Dicots owned ten exons. The genes *AtPDIL1-1 *of Arabidopsis and *OsPDIL1-3 *of rice contained nine exons, most probably they were produced by the deletion of the 7^th ^and 2^nd ^introns, respectively (Additional file 8). The genomic organization observed in the *PDI *genes of the angiosperms is very old and has been conserved during evolution; in fact eight of the nine exons of the moss *P. patens *gene *PpPDIL1-1 *were similar to those in the angiosperms' genes, suggesting that this genomic organization was already present before their diversification. The close sequence homology and the similar exon/intron structure, suggests that *AtPDIL1-2 *of Arabidopsis, *OsPDIL1-1 *of rice and *TaPDIL1-1 *of wheat represent the orthologous genes, whereas *AtPDIL1-1*, *OsPDIL1-2 *and *OsPDIL1-3 *would be paralogous genes produced by duplication and diversification events. All the genes of the first phylogenetic group have in both active sites the conserved tetrapeptide -CGHC-.

The genomic structure of the genes in the second phylogenetic group (*TaPDIL2-1 *of wheat; *OsPDIL2-1 *of rice; *AtPDIL2-1 *and *AtPDIL2-2 *of Arabidopsis) and of the third group (*TaPDIL3-1 *of wheat; *OsPDIL3-1 *of rice; *AtPDIL3-1 *and *AtPDIL3-2 *of Arabidopsis) included 12 exons. The exons of the genes in the second group were highly conserved, except for the first and last exons and an insertion of three nucleotides in the third exon of *OsPDIL2-1*, whereas the genes of the third group were less conserved, in terms of intron positions and exon lengths (Additional file 8 and Figure 5). Besides the terminal ones, the exons of genes in the third group showed the most relevant divergence from the corresponding exons of the genes of the second group (6, 7, 9, 10 and 11), whereas exons 2, 3, 4, 5, 8 and 10 were highly conserved between the two groups. The comparison of the genomic organization supports the common origin of the genes belonging to the phylogenetic groups 2 and 3 and is consistent with their close clustering obtained by phylogenetic analysis (Fig 1 and Additional file 6) and with their conserved domain architecture (Fig 2). The genes of the second and third group showed a tightly conserved location of the sequences encoding the tetrapeptide active sites, which in wheat were 200 bp from the end of the first exon and 120 bp from the end of the tenth exon; the amino acid sequences of the tetrapeptides were less conserved, especially among proteins encoded by the genes of the third group. The most conserved sequence -CGHC- was present in the C-terminal active site of the four proteins in the second group and in the N-terminal site of the proteins TaPDIL2-1 and AtPDIL2-2, whereas the N-terminal tetrapeptide of OsPDIL2-1 was -CAHC- and that of AtPDIL2-1 was -CGAC-. The C-terminal active site in the third group proteins was -CVNC- in AtPDIL3-1, -CINC- in AtPDIL3-2 and -CVDC- in TaPDIL3-1 and OsPDIL3-1; the N-terminal tetrapeptide was -CARS- in both Arabidopsis proteins and -CERS- in the proteins of wheat and rice. In *P. patens *there were six PDI-like genes in the second phylogenetic group and two in the third; their genomic structure was very diversified in exon number: *PpPDIL2-4 *in the second group and *PpPDIL3-1 *in the third group had the typical structure of the genes belonging to these groups consisting of 12 exons, whereas the other *P. patens *genes showed a lower number of exons, in particular *PpPDIL2-1 *and *PpPDIL2-2 *owned a single exon (Additional file 8).

The fourth phylogenetic group included a single gene of wheat (*TaPDIL4-1*) and Arabidopsis (*AtPDIL4-1*) and two of rice (*OsPDIL4-1 *and *OsPDIL4-2*); the three monocot genes had 11 exons, whereas the of Arabidopsis one had 10 exons (Additional file 9). Except the terminal ones, the exons were highly conserved in all the four genes. Since the *P. patens *gene *PpPDIL4-1 *had 11 exons like the genes of wheat and rice, most probably the ancestral gene of the fourth group had 11 exons and one of them got lost during Arabidopsis evolution. All the active sites of the IV group proteins had the conserved tetrapeptide -CGHC-.

The fifth group contained a single gene of wheat (*TaPDIL5-1*) and rice (*OsPDIL5-1*), whereas Arabidopsis was represented by two paralogous genes (*AtPDIL5-1 *and *AtPDIL5-2*). All the genes in this phylogenetic group, including the *P. patens *gene (*PpPDIL5-1*), enclosed nine exons whose length, except for the first and last ones, were perfectly conserved across the analysed species; also the typical tetrapeptide -CGHC- was conserved in all the protein active sites.

The genes in the sixth phylogenetic group, *AtPDIL6-1*, *OsPDIL6-1 *and *TaPDIL6-1*, presented a conserved genomic structure including four exons, whereas the moss *P. patens PpPDIL6-1 *exibited a single exon (Additional file 8). Possible explanations are either intron colonization after the separation of angiosperms and bryophytes or intron loss during *P. patens *evolution [97]. The single tetrapeptide active site of these proteins was the typical -CGHC- in the moss, and the slightly modified -CKHC- in the three angiosperm.

The seventh group included two genes of wheat (*TaPDIL7-1 *and *TaPDIL7-2*), two of rice (*OsPDIL7-1 *and *OsPDIL7-2*) and a single gene of Arabidopsis (*AtPDIL7-1*): they had five exons and their protein products had the single typical tetrapeptide -CGHC-; the moss gene *PpPDIL7-1 *had four exons and a modified tetrapeptide -CKHC-.

The eighth phylogenetic group contained two genes of Arabidopsis (*AtPDIL8-1 *and *AtPDIL8-2*) and one of rice (*OsPDIL8-1*), wheat (*TaPDIL8-1*) and moss (*PpPDIL8-1*); all the genes included 15 very well conserved exons. The proteins belonging to this phylogenetic group presented a non characteristic tetrapeptide sequence at their single active site: -CYWS- in *AtPDIL8-1*, *OsPDIL8-1 *and *TaPDIL8-1*, -CYWC- in *AtPDIL8-2 *and -CPWS- in *PpPDIL8-1*. The well conserved structure of these genes, even in the moss, suggests that this complex genomic organization was already present in the ancestral gene of the common ancestor of angiosperms and bryophytes.

### Expression patterns of PDI and PDI-like genes in wheat tissues

The relative and absolute quantification of the transcripts of nine PDI and PDI-like genes in different tissues and developmental stages (data set n. 1), are reported in Additional file 9. In order to make the graphs more readable, the nine genes were split into two groups on the basis of their phylogenetic and structural relationships. The first group included five genes belonging to the first major clade of the plant PDI family (*TaPDIL1-1*, *TaPDIL2-1*, *TaPDIL3-1*, *TaPDIL7-1 *and *TaPDIL7-2*), the second group comprised two genes of the second major clade (*TaPDIL4-1 *and *TaPDL5-1*) and two additional genes (*TaPDIL6-1 *and *TaPDIL8-1*) coding for proteins with a single thioredoxin domain. The nine genes were constitutively expressed in all the analysed tissues, even though their transcription levels were highly variable. *TaPDIL1-1 *and *TaPDIL2-1 *were highly expressed in immature caryopses (Seeds2 collected 10 DPA in Additional file 9A and 9B); the estimated absolute number of transcripts per μg of total RNA exceeded 900,000 copies for *TaPDIL1-1 *and 300,000 copies for *TaPDIL2-1 *(Additional file 9B). The expression level of *TaPDIL1-1 *remained relatively high also during later stages of seed development, with absolute amount of transcripts of about 200,000 copies in 38 DPA caryopses (Seeds 7, Additional file 9B). The transcription level of the other seven PDI-like genes in all the tested tissues and that of *TaPDIL1-1 *and *TaPDIL2-1 *in the remaining ten tissues did not exceed 120,000 copies per μg of total RNA (Additional file 11B and D). All the wheat genes of the PDI family showed a broad range of expression across the analysed tissues, as expected for genes accomplishing key metabolic functions and as demonstrated for the PDI family members in all studied plant species [20,25-28,98]. The highly diversified expression rates and patterns exhibited by the analysed genes are easily explained by the need of their spatial and temporal regulation requested to accomplish the metabolic role of the PDI and PDI-like proteins in specific tissues and developmental stages. The very high transcription of *TaPDIL1-1 *and *TaPDIL2-1 *detected in the caryopses is consistent with their proposed role during the seed development, in particular for the correct synthesis and accumulation of seed storage proteins and starch, as discussed later.

**Figure 6:**
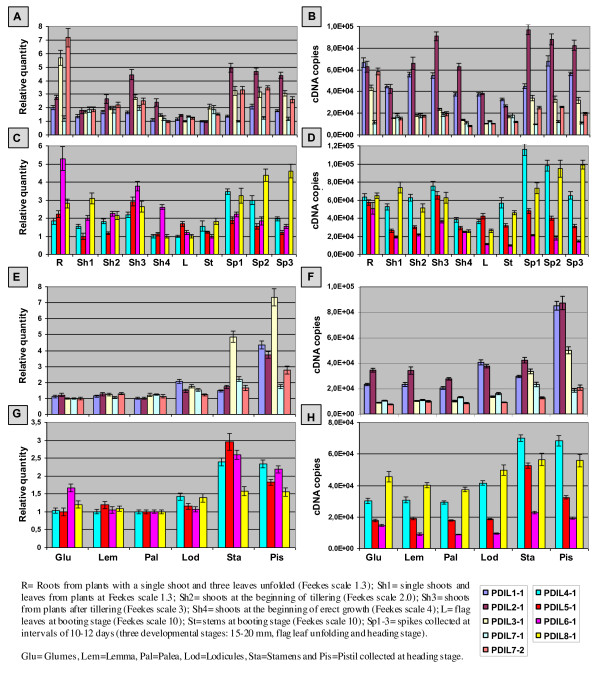
**Expression analysis of PDI and PDI-like genes in different tissues and floral organs**. Relative (A, C, E and G) and absolute (B, D, F and H) quantification of the expression level of nine PDI and PDI-like genes in 10 tissues or developmental stages of wheat (A, B, C and D) and in wheat floral organs from fully emerged spikes (E, F, G and H). 20 cDNA pools (two biological replicates, 10 plant samples; A, B, C and D) and 12 cDNA pools (two biological replicates, 6 floral organ samples; E, F, G and H) were tested in triplicate and normalized using the geometric average of the relative expression of the two reference genes encoding Cell division control protein and ADP-ribosylation factor. The relative expression levels of the nine genes were referred to that of a calibrator set to the value one, which was represented by the tissue (A and C) or floral organ (E and G) with the lowest expression. The absolute expression levels of the nine genes were expressed as number of cDNA copies per μg of reverse transcribed total RNA (B, D, F and H). Normalized values of relative and absolute expressions of the nine genes are given as average ± SD.

Caryopses were excluded from the second data set (Figure 6A-D), since the very high transcription of *TaPDIL1-1 *and *TaPDIL2-1 *in this sample flattened and masked the expression variation among the remaining tissues (see graphs in Additional file 11). Three groups were recognized among the nine genes considering the average expression level in the ten samples of the second data set: three genes (*TaPDIL2-1*, *TaPDIL4-1 *and *TaPDIL8-1*) with high (more than 60,000 copies); two genes (*TaPDIL1-1 *and *TaPDIL5-1*) with medium (between 40,000 and 50,000 copies) and four genes (*TaPDIL3-1*, *TaPDIL6-1*, *TaPDIL7-2 *and *TaPDIL7-1*) with low expression (below 25,000 copies).

The expression of the gene encoding the typical PDI (*TaPDIL1-1*) was quite constant in all the ten tissues analysed, the highest transcription levels being detected in the middle stage of spike development (Spikes2) and in roots (Figure 6A and 6B). The transcription level of *TaPDIL2-1 *was very high in spikes (in particular during the early stages of development) and significantly higher than that of *TaPDIL1-1*, whereas its expression was moderate in roots and very low in stems and leaves of plants at booting stages (Figure 6A and 6B). The transcription rate of *TaPDIL2-1 *increased during the vegetative growth and reached a maximum at the tillering stage (Shoots3); moreover, its expression during shoot development was the highest among the nine genes. The protein encoded by the Arabidopsis gene *AtPDIL2-1 *(*AtPDIL1-3 *in [20]), homologous to *TaPDIL2-1*, was detected in the stromal-starch interface of leaf chloroplasts, suggesting its involvement in the starch synthesis within leaf tissues and possibly intervening in the posttranslational redox regulation of the activity of ADP-glucose pyrophosphorylase (AGPase) [99]. Among the proteins encoded by the PDI and PDI-like genes that have been characterized in soybean [25-28]; GmPDIL-2, the soybean orthologous to the Arabidopsis AtPDIL2-1, in agreement with its potential role in starch biosynthesis was found highly expressed during all the leaf developmental stages and the only protein of the PDI family highly expressed in fully developed leaves [26]. The high expression of *TaPDIL2-1*, detected not only during shoots development but also in developing spikes and caryopses of wheat, seems congruent with its metabolic role, since active starch synthesis takes place in all these tissues.

The expression patterns of *TaPDIL4-1 *and *TaPDIL8-1 *were very similar to that of *TaPDIL2-1*, but the transcription of *TaPDIL8-1 *was lower in the early stages of spike development and increased during middle to late stages until heading time (Figure 6C and 6D). The possible involvement of *TaPDIL4-1 *in the development of the vegetative and reproductive meristems is suggested by its strong expression during early stages of spike, shoot and root development. The orthologous gene of Arabidopsis *AtPDIL4-1 *(*AtPDIL2-1 *in [20]) of the same group IV showed a similar expression pattern in the analysed tissues [100]. The functional analysis of transgenic Arabidopsis plants showed its involvement during seed set and ovule development.

*TaPDIL3-1 *encoded a protein whose structure was similar to that of TaPDIL2-1; however its expression was much lower in most analysed tissues, but much higher in roots (Figure 6). Two ER resident proteins of soybean (GmPDIL-3a and GmPDIL-3b), encoded by genes orthologous to *TaPDIL3-1*, were ubiquitously expressed in the analysed tissues and their expression increased in the cotyledons during seed maturation; however, as discussed previously, they did not exhibit any oxidoreductase or molecular chaperone activity and their metabolic role is not clear [28]. As observed for *TaPDIL3-1*, the expression of *TaPDIL7-2 *and *TaPDIL6-1 *was higher in roots than in other tissues, but comparable, or even slightly lower, to that of the genes included in the high and intermediate expression groups (*TaPDIL2-1*, *TaPDIL4-1*, *TaPDIL8-1 *and *TaPDIL1-1*). The expression levels of *TaPDIL3-1 *and *TaPDIL7-2 *were relatively higher also in spikes, whereas the lowest amount of transcripts was detected in vegetative tissues, such as leaves, stems and shoots. Instead, the transcription level of *TaPDIL5-1 *was relatively higher in shoots after tillering (Shoots3) and at the beginning of erect growth (Shoots4) than in spikes (Figure 6C and 6D). Finally, the expression of *TaPDIL7-1 *did not show any remarkable variation among the ten tissues analysed, a slightly higher level of transcription was observed in shoots collected from plants at the stage of three unfolded leaves (Shoots1), at the beginning of tillering (Shoots2) and with formed tillers (Shoots3) and in stems (Figure 6C and 6D).

### Expression patterns of PDI and PDI-like genes in wheat floral organs

The expression of the nine PDI and PDI-like genes was also compared in six single floral organs from fully emerged spikes (Figure 6E-H). Generally, the transcription of the nine genes was lower in the flower vegetative organs (glumes, lemma, palea and lodicules), than in the male and female reproductive organs (stamens and pistil); however the absolute copy number of transcripts varied considerably. The highest average absolute expression levels were detected for *TaPDIL2-1*, *TaPDIL4-1 *and *TaPDIL8-1 *(more than 40,000 copies/μg RNA); intermediate for *TaPDIL1-1 *and *TaPDIL5-1 *(between 25,000 and 40,000 copies); lowest for *TaPDIL3-1*, *TaPDIL6-1*, *TaPDIL7-1 *and *TaPDIL7-2 *(between 10,000 and 25,000 copies). The expression pattern of *TaPDIL1-1 *and *TaPDIL2-1 *was very similar and their transcription level in the pistil was about 3 times higher than in other floral organs (Figure 6E and 6F); also the expression of *TaPDIL7-2 *was higher in the pistil than in other floral organs, but lower than *TaPDIL1-1 *and *TaPDIL2-1*. *TaPDIL3-1 *expression in stamens and pistil was 5-7 times higher than in glumes, lemma and palea, and 3-4 times higher than in lodicules. Also *TaPDIL4-1*, *TaPDIL5-1 *and *TaPDIL6-1 *were more expressed in both the reproductive floral organs, but their transcription rate was only twice higher than in vegetative floral organs (Figure 6G and 6H). Finally, the expression of *TaPDIL7-1 *and *TaPDIL8-1 *was only slightly higher in stamens and pistil than in the flower vegetative organs.

The expression of all the analysed genes was higher in the reproductive than in the vegetative floral organs, most probably for accomplishing the more specialised developmental program and more intense metabolic activity [101]. To our knowledge the only reported expression analysis for a PDI protein in different floral organs has involved the typical PDI (PDI5) of Arabidopsis which is encoded by *AtPDIL1-1*, homologous to the wheat gene *TaPDIL1-1 *[102]. PDI5 was preferentially expressed in all gynoecial tissues including the ovary, the septum and the ovules, where it was located in the ER of endothelial cells; expression of PDI5 was also detected, although at lower level, in stamens and petals. Loss of PDI5 leads to premature initiation of PCD (Programmed Cell Death) in endothelial cells during embryo development and to formation of fewer and often non viable seeds. Consequently, Ondzighi et al. [102] have proposed that, beside its well known functions, the typical PDI of Arabidopsis is also required for proper embryogenesis and temporal progression of PCD by chaperoning and inhibiting Cys proteases during their trafficking via Golgi to vacuoles, before the initiation of PCD of endothelial cells.

### Expression patterns of PDI and PDI-like genes in developing caryopses

**Figure 7:**
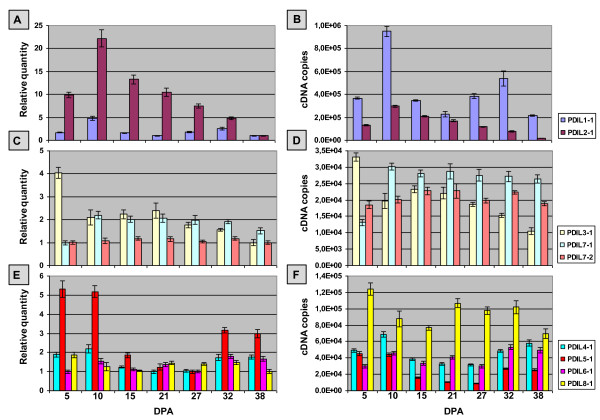
**Expression analysis of PDI and PDI-like genes in developing caryopses**. Relative (A, C and E) and absolute (B, D and F) quantification of the expression level in developing caryopses collected between 5 and 38 DPA (days post anthesis) of nine PDI and PDI-like genes. The 14 cDNA pools (two biological replicates, seven developing caryopsis samples) were tested in triplicate and normalized using the geometric average of the relative expression of the two reference genes encoding Cell division control protein and ADP-ribosylation factor. The relative expression levels of the nine genes (A, C and E) were referred to that of a calibrator set to the value one, which was represented by the developmental stage with the lowest expression. The absolute expression levels of the nine genes were expressed as number of cDNA copies per μg of reverse transcribed total RNA (B, D and F). Normalized values of relative and absolute expressions of the nine genes are given as average ± SD.

The transcription levels of the nine genes of the wheat PDI family were investigated in a series of seven developmental stages of caryopses collected at 5-6 days intervals from 5 to 38 days post anthesis (DPA) (Figure 7A-F). The analysed caryopses covered the stages of endosperm development from cellularisation to desiccation, or physiological maturity [103,104]. Analyses by qRT-PCR indicated a considerable variation of transcription rate between the nine genes. The highest average absolute amount of transcripts in the seven seed samples was detected for *TaPDIL1-1 *(433,386 copies), *TaPDIL2-1 *and *TaPDIL8-1 *(both about 100,000 copies); *TaPDIL4-1 *and *TaPDIL6-1 *had an intermediate expression (about 40,000 copies); the lowest expression was exhibited by *TaPDIL3-1*, *TaPDIL5-1*, *TaPDIL7-1 *and *TaPDIL7-2*, whose average absolute amount of transcripts was comprised in the range of 20,000-25,000 copies per μg of total RNA.

The most expressed genes *TaPDIL1-1*, *TaPDIL2-1 *and *TaPDIL8-1 *displayed divergent expression patterns during grain development. *TaPDIL1-1 *expression was higher in the very early stages of seed development (highest level 10 DPA), then decreased steeply between 15 and 21 DPA, during the transition from the endosperm cell division (cellularisation) to the start of grain filling (the deposition of starch and gluten proteins), then increased again until 32 DPA, when the deposition of seed storage reserves decreased and seeds started desiccating (Figure 7A and 7B); the lowest level of transcription was detected at the physiological maturity (38 DPA). Also the expression of *TaPDIL2-1 *was higher during the early stages of seed development (10 DPA), then decreased gradually, reaching the lowest level at the physiological maturity (Figure 7A and 7B). The expression pattern of *TaPDIL8-1 *was more complex: its transcription was very high five DPA, decreased 10 and 15 DPA, increased again 21 DPA, remaining at the same level 32 DPA, then reached the lowest level at physiological maturity 38 DPA (Figure 7E and 7F). The expression patterns of *TaPDIL4-1 *and *TaPDIL5-1 *were fairly similar to that of the typical PDI (*TaPDIL1-1*), although their overall transcription rate was much lower. They were highly expressed during the early stages of seed development (5-10 DPA), moderately expressed at the end of the grain filling and at physiological maturity (32-38 DPA), and expressed at very low level during grain filling (15-27 DPA). A similar behaviour was also observed for *TaPDIL6-1*, with the only difference that its transcription level was slightly higher during late (32-38 DPA) than early stages (5-10 DPA) of grain development. The expression of *TaPDIL3-1 *was very high 5 DPA, then decreased rapidly 10 DPA and remained at the same level until 21 DPA, then decreased gradually until physiological maturity (38 DPA) (Figure 7C and 7D). Finally, the transcription rate of *TaPDIL7-1 *increased about twice from 5 to 10 DPA, then remained stable for the remainder of the time-course studied, whereas no significant variation was detected in the expression of its paralogous gene *TaPDIL7-2 *in the seven seed samples analysed.

Proteins of the PDI family are important components of the machinery that assists in the folding, assembling and sorting of secretory proteins via the ER; since wheat seeds contain a variety of secretory proteins, their folding could be assisted by PDI proteins. Apart from a low amount of proteases, hydrolases and α-amylase inhibitors, the seed storage proteins (prolamins or gluten proteins) represent 80-90% of total seed proteins; consequently the very high seed expression of *TaPDIL1-1*, *TaPDIL2-1 *and *TaPDIL4-1*, whose protein products contain two thioredoxin active domains, may be related to their role in the developing wheat endosperm. The study of the synthesis and deposition of the major storage components during wheat seed development has shown that the accumulation of gluten proteins and starch takes place essentially in parallel fashion [105]. Both processes increase steadily between 12 and 35 DPA and keep on constantly until 42 DPA, when protein accumulation virtually ceases; a dramatic increase of large glutenin polymers takes place during grain desiccation [105]. Present data on expression analysis showed that the mRNA level of some PDI genes such as *TaPDIL1-1*, *TaPDIL2-1*, *TaPDIL4-1*, and *TaPDIL5-1*, whose encoded proteins contain two active thioredoxin domains, increased dramatically during the early stages of seed development (5-10 DPA), then declined steadily during the major part of the grain filling period (15-27 DPA), indicating that the temporal expression of these PDI genes was not tightly co-ordinated with the expression of storage proteins, starting earlier in grain development and reaching a maximum before the period of highest synthesis of gluten proteins. A key role of the wheat PDI and PDI-like proteins in the folding and deposition of wheat storage proteins cannot reliably be assumed merely on the basis of the present expression data, which,. however, point to their involvement at some early stage of protein processing and protein body formation or to a more general housekeeping role in the processing of secretory proteins. For *TaPDIL1-1*, *TaPDIL4-1 *and *TaPDIL5-1 *it is noteworthy the presence of a second peak of transcripts detected by qRT-PCR between 27 and 32 DPA, when the deposition of seed storage reserves decreased and seeds started desiccating. Seemingly the PDI proteins encoded by these genes may play an important role in later stages of seed development, when there is a dramatic increase of large gluten polymers stabilised by the formation and/or the rearrangement of inter-chain disulphide bonds [105].

The abundance of *TaPDIL2-1 *transcripts found in the developing caryopses could also be related to the starch synthesis, as shown for other PDI-like genes of the second phylogenetic group in vegetative tissues of other plant species [99]. Moreover, it is interesting the similarity between its expression pattern and that of the gene encoding the small subunit of the cytosolic AGPase (ADP-glucose pyrophosphorylase) of wheat, which was expressed at high levels early in endosperm development (6-10 DPA) and then declined steadily until physiological maturity [105]. Since this enzyme catalyzes the synthesis of ADPglucose, the precursor of the starch synthesis, TaPDIL2-1 might participate to the redox regulation of the cytosolic AGPase in the endosperm of developing wheat seeds.

Like wheat endosperm, soybean cotyledons contain large amounts of seed storage proteins such as glycinin and β-conglycinin, which are synthesized and folded within the ER during seed development. As previously discussed, recent studies indicated that at least four (GmPDIL-1, GmPDIL-2, GmPDIS-1 and GmPDIM) of the seven characterized soybean proteins of the PDI family would be involved in the folding of storage proteins, acting both as thiol-oxidoreductase and as a molecular chaperone [25-28]. The transcripts of *GmPDIL-2 *remained almost stable during seed development [27], whereas the mRNA levels of *GmPDIL-1*, *GmPDIS-1 *and *GmPDIM *were much higher in the very early stage of seed development, as their orthologous wheat genes *TaPDIL1-1*, *TaPDIL4-1 *and *TaPDIL5-1*, then declined steadily from the middle to the late stages of seed development [25-27]. Their transcription rate, however, did not correlate with the expression level of the encoded proteins. In fact the amount of the proteins GmPDIL-1, GmPDIS-1 and GmPDIM were relatively high in the early stages of seed development and persisted almost at the same level (GmPDIL-1 and GmPDIM) or increased (GmPDIS-1) until seed maturation [25-27]. As a matter of fact, the amount of the three proteins during the late stages of seed development would be regulated by a post-transcriptional mechanism which would control the differential expression of the PDI proteins. In fact the GmPDIL-1, GmPDIS-1 and GmPDIM proteins persisted at very high levels during the late stages of seed maturation, seemingly for their importance in the folding of seed storage proteins, whereas at the same stages GmPDIL-2 and GmPDIS-2 (paralogous to GmPDIS1) exhibited a low level of expression. Future studies on the expression levels of wheat PDI and PDI-like proteins during seed development and their comparisons with the levels of the corresponding transcripts will be important to investigate the mechanisms involved in the temporal regulation of their encoding genes and to elucidate their role and importance in the folding of seed storage proteins and specifically in the formation of high molecular weight aggregates.

## Conclusions

PDI and PDI-like proteins are responsible for multiple metabolic functions, including secretory protein folding, chaperone activity and redox signalling. Specific knowledge of their diversified roles has resulted from an increasing number of studies, most of them involving mammals, whereas in plants the knowledge on the structural and functional features of this versatile group of proteins and of their encoding genes is much less extensive. The purpose of our research was the characterization of the genes encoding PDI and PDI like proteins in wheat and the comparison of their structure and expression with those of homologous genes isolated in other plant species. Former studies in wheat had been restricted to the characterization of the genes encoding the typical PDI, which is of special interest for its involvement in determining the bread making quality and flour technological properties. The interest of extending the study to additional members of the PDI gene family is related to their potentially relevant metabolic functions, as well as to the knowledge of their molecular evolution in a polyploid context. Despite the recent data on the complexity and diversity of the PDI gene family in plants, there are still a number of unanswered questions concerning cell location and physiological functions of their protein products. For each of them it will be necessary to determine whether they have overlapping and redundant or separate and specific target substrates and whether they act independently or by interacting with other proteins in a redox chain. In wheat, functional analysis will be necessary to understand the physiological role of each gene product of the PDI family, in particular their involvement in the folding, transport and deposition of the seed storage proteins. Besides ultrastructural and biochemical studies and the information on the metabolic role in other plant species of phylogenetically related genes, the functional analysis will require the selective silencing of the PDI and PDI-like genes in wheat plants and the characterization of the regulatory motifs through the expression studies of the progressive deletions of their promoters. The comprehensive structural and expression characterisation of the complete set of *PDI *and *PDI*-like genes of wheat presented in this study may represent a basis for the functional characterisation of this gene family in the hexaploid context of bread wheat.

## Authors' contributions

MC and ED designed experiments and drafted the manuscript. ED and APD performed sample preparation and experimental procedures for the characterisation of the PDI-like genes. ARP performed sample preparation and experimental procedures for expression analyses by qRT-PCR. MC, ED, ARP and OAT performed data analysis. All the authors discussed the results. EP and OAT revised critically the manuscript. EP and MC provided financial support to the study. All authors read, discussed and approved the final manuscript.

## Supplementary Material

Additional file 1 **Primer pairs used for the isolation of the full-length cDNA and genomic sequences of eight novel PDI-like genes**. Click here for file

Additional file 2 **Accession numbers of the full-length cDNA and genomic sequences deposited in the DDBJ/EMBL/GeneBank nucleotide sequence databases**. A code of two letters (Ta = Triticum aestivum) followed by the suffix PDIL and by an Arabic number indicating the corresponding phylogenetic group was assigned to each sequence. Multiple sequences clustering into the same subfamily were designed by an additional number (1-2). Multiple clones are indicated with the corresponding letter a, b or c. Click here for file

Additional file 3 **Protein ID of the different PDI-like genes used in the phylogenetic analysis**. Click here for file

Additional file 4 **Primer pairs used in Southern analyses and corresponding amplification product size**. Click here for file

Additional file 5 **Expression analyses by qRT-PCR of the nine wheat PDI and PDI-like genes**. This additional file describes the experimental procedures for the absolute and relative quantification by qRT-PCR of the expression levels of the nine PDI and PDI-like genes. In particular it reports: a) the list of primer pairs used in qRT-PCR analyses; b) the specificity of qRT-PCR amplifications; c) the characteristics of the standard curves used for estimating the absolute copy number of cDNAs corresponding to the nine PDI and PDI-like genes; d) the method used for the normalizazion of absolute and relative data. Click here for file

Additional file 6 **Phylogenetic tree based on the deduced amino acid sequences of 108 plant PDI-like genes**. The phylogenetic tree shows the relationships between the deduced amino acid sequences of the PDI and PDI-like genes of different plant species: nine of wheat, 13 of *A. thaliana *(At), 12 of *P. trichocarpa *(Pt), 10 of V. vinifera (Vv), 21 of *G. max *(Gm), 12 each of *Z. mais *(Zm) and *O. sativa *(Os), 14 of *P. patens *(Pp) and five of *C. reinhardtii*. Multiple alignment of the sequences was performed by ClustalX 1.83 software and the phylogenetic tree was constructed by the neighbour-joining (NJ) method and evaluated by bootstrap analysis (PHYLIP version 3.6). The numbers on the main branches indicate bootstrap percentages for 1,000 replicates. The PDI-like sequences of groups VI and VIII were used as outgroups, due to their high diversification from the other subfamilies. The two major clades (I and II) and the eight phylogenetic groups (I-VIII) indentified in the plant PDI family are highlighted with curly and square brackets, respectively. Click here for file

Additional file 7 **Chromosome location and syntenic relationships of the PDI-like genes of wheat and rice**. Chromosome location of wheat PDI-like genes determined through Southern or PCR analysis (this study), Chromosome location and position of the orthologous rice genes as well as the flanking Wheat Bin Mapped Marker mapped onto Rice Pseudomolecules http://rice.plantbiology.msu.edu/cgi-bin/gbrowse/rice/ are reported as in Release 6.1 of the MSU Rice Genome Annotation (Osa1 June 3 2009). For each Wheat Bin Mapped Marker the accession number of the corresponding probe used in the wheat deletion mapping project, wheat chromosome arms involved as well as their alignment position on the rice Pseudomolecules are also reported. Corresponding wheat chromosome bin information is not reported but can be retrieved based on probe information http://wheat.pw.usda.gov/cgi-bin/westsql/map_locus.cgi. Click here for file

Additional file 8 **Genomic structure of the PDI-like genes of *T. aestivum, O. sativa*, *A. thaliana *and *P. patens***. Exon-intron structure of the PDI and PDI-like genes of wheat, Arabidopsis, rice and *P. patens *are reported. Exons (yellow boxes) and introns (blue boxes) are reported in their original 5'-3' orientation, their length is reported in bp. Only the ORF sequences have been indicated, whereas the 5' and 3' UTRs have been omitted. ORFs (bp and aa), number of exons, tetrapeptide sequences at the N and C-terminal thioredoxin-like active site and C-terminal tetrapeptide sequences have also been reported. Numbers in bold indicate the size of the exons comprising the thioredoxin-active site. Click here for file

Additional file 9 **Expression analysis of PDI and PDI-like genes in different tissues and developing caryopses**. Relative (A and C) and absolute (B and D) quantification of the expression level of nine PDI and PDI-like genes in 12 tissues and developmental stages of wheat. The 24 cDNA pools (two biological replicates, 12 plant samples) were tested in triplicate and normalized using the geometric average of the relative expression of the two reference genes encoding Cell division control protein and ADP-ribosylation factor. The relative expression levels of the nine genes were referred to that of a calibrator set to the value one, which was represented by the tissue with the lowest expression (A and C). The absolute expression levels of the nine genes were expressed as number of cDNA copies per mg of reverse transcribed total RNA (B and D). Normalized values of relative and absolute expressions of the nine genes are given as average ± SD. Click here for file
