# Supplementary material for: The Protein Disulfide Isomerase gene family in bread wheat (T. aestivum L.)
Source: BMC Plant Biol. 2010 Jun 3;10:101. doi: 10.1186/1471-2229-10-101 (PMC3017771; doi:10.1186/1471-2229-10-101)
Supplement: Additional file 1 — Primer pairs used for the isolation of the full-length cDNA and genomic sequences of eight novel PDI-like genes. [file 1471-2229-10-101-S1.PDF]

Gene	Forward primer	Reverse primer
TaPDIL2-1	5’-GGCGGATCCATTTCCACTC-3’	5’-TCAGCATACACCTCCAACGG-3’
TaPDIL3-1	5’-GCCATTAGCACCTCACCA-3’	5’-ATGGCTACTGCGTAACCGT-3’
TaPDIL4-1	5’-GTGCAAGAAGAACAGGTGCC-3’	5’-CCGCTAAACTTTCACTGCCA-3’
TaPDIL5-1	5’-TCGATCGCCACCATCTAGC-3’	5’-CCACCTTGCACATCAGAGCTT-3’
TaPDIL6-1	5’-CTCCGATCGAGCCATGGATC-3’	5’-TAAGCCCAAGCCCGCAAA-3’
TaPDIL7-1	5’-CAGACGCGGCGGAAGATCGAA-3’	5’-TGATACACCATATAACCCTCGCA-3’
TaPDIL7-2	5’-CGTAGTGTTCCACGCAGCTA-3’	5’-GACATGAAATCGCTAAACAGAGG-3’
TaPDIL8-1	5’-GGCACACCTTTCGAAACG-3’	5’-GACTGGTGACAAGATCAGGATGA-3’
