# Supplementary material for: The Protein Disulfide Isomerase gene family in bread wheat (T. aestivum L.)
Source: BMC Plant Biol. 2010 Jun 3;10:101. doi: 10.1186/1471-2229-10-101 (PMC3017771; doi:10.1186/1471-2229-10-101)
Supplement: Additional file 2 — Accession numbers of the full-length cDNA and genomic sequences deposited in the DDBJ/EMBL/GeneBank nucleotide sequence databases. A code of two letters (Ta = Triticum aestivum) followed by the suffix PDIL and by an Arabic number indicating the corresponding phylogenetic group was assigned to each sequence. Multiple sequences clustering into the same subfamily were designed by an additional number (1-2). Multiple clones are indicated with the corresponding letter a, b or c. [file 1471-2229-10-101-S2.PDF]

Gene	Accession number cDNA	Accession number gene
TaPDIL2-1	clone a) FN555316	FN555176
TaPDIL3-1	clone a) FN555317	FN555309
TaPDIL4-1	clone a) FN555318<br>clone b) FN555319	FN555310
TaPDIL5-1	clone a) FN555320<br>clone b) FN555321	FN555311
TaPDIL6-1	clone a) FN555322<br>clone b) FN555323	FN555312
TaPDIL7-1	clone a) FN555324<br>clone b) FN555325<br>clone c) FN555326	FN555313
TaPDIL7-2	clone a) FN555327<br>clone b) FN555328	FN555314
TaPDIL8-1	clone a) FN555329	FN555315
