# Supplementary material for: The Protein Disulfide Isomerase gene family in bread wheat (T. aestivum L.)
Source: BMC Plant Biol. 2010 Jun 3;10:101. doi: 10.1186/1471-2229-10-101 (PMC3017771; doi:10.1186/1471-2229-10-101)
Supplement: Additional file 4 — Primer pairs used in Southern analyses and corresponding amplification product size. [file 1471-2229-10-101-S4.PDF]

Gene	Forward primer	Reverse primer	(bp)
TaPDIL2-1	5’-CGTCAAAGTTGTTGTTGGCAA-3’	5’-CAGCATACACCTCCAACGGC-3’	553
TaPDIL3-1	5’-TTGCAGTTTGTGGAGCTTAAC-3’	5’-ATGGCTACTGCGTAACCGT-3’	929
TaPDIL4-1	5’-TCTGACCGAGGAAACCTTTGA-3’	5’-CCGCTAAACTTTCACTGC-3’	785
TaPDIL5-1	5’-TTGTGGAGTTCTTTGCACCG-3’	5’-CCACCTTGCACATCAGAGCTT-3’	917
TaPDIL6-1	5’-TCCCTACCCATCTCCTGCTG-3’	5’-AGTTAGCCACAAGCGCCA-3’	555
TaPDIL7-1	5’-TTGCCAAAGTAAATGCCGA-3’	5’-TTGCTGACAGAGAAACGGC-3’	1119
TaPDIL7-2	5’-CGTAGTGTTCCACGCAGCTA-3’	5’-CTGGAGCCGGAAACTACTACAC-3’	1331
TaPDIL8-1	5’-AAGCGACCTGCTCCAATGAC-3’	5’-CCCAACATTCAGACCAACCC-3’	733
