# Supplementary material for: The Protein Disulfide Isomerase gene family in bread wheat (T. aestivum L.)
Source: BMC Plant Biol. 2010 Jun 3;10:101. doi: 10.1186/1471-2229-10-101 (PMC3017771; doi:10.1186/1471-2229-10-101)

Additional file 5 – Expression analyses by qRT-PCR of the nine wheat PDI and PDI-like genes.

Total RNA was extracted using the TRIzol reagent (Invitrogen) according to manufacturer's instructions, whereas from caryopses it was isolated by a LiCl based method. The resulting RNA was treated with RNase-free DNase I (Promega) according to the manufacturer's protocol. Following digestion, nucleotides were removed from RNA using a G50 sepharose buffer exchange column (Amersham). Absence of genomic DNA contamination in DNase I-treated samples was checked by PCR using a primer pair (5'-CTTATGCAATCCAATGATGG-3' and 5'-TCTATGGTTCTGAAGAGGACC-3') designed to amplify an intron sequence of a gene encoding typical PDI and located on chromosome 4A [1]. When a single DNase treatment did not completely remove interfering genomic DNA, a second DNase incubation was performed to eliminate any detectable DNA. Synthesis of cDNA from the DNase I treated RNA was only performed when the genomic control amplifications scored negative. RNA concentration and integrity were checked with a UV/VIS spectrophotometer Lambda 3B (Perkin Elmer) before and after DNase I digestion. The quality of RNA samples was also assessed by electrophoresis on 1% formaldehyde agarose gels. First-strand cDNA was synthesized from 3 μg of RNA by the Expand™ Reverse Transcriptase (ROCHE) and the resulting cDNA was diluted one-fifth for qRT-PCR analyses.

A set of candidate genes previously evaluated for their expression stability in different wheat tissues [2] were initially selected to identify the most suitable reference genes in our experimental conditions. The six candidate reference genes encoded the following proteins: ADP-ribosylation factor (Unigene Cluster Ta2291; DFCI contig TC278558), Ubiquinol-cytochrome C reductase (Unigene Cluster Ta4045; DFCI contig TC350975), Cell division control protein belonging to the AAA-superfamily of ATPases (Unigene Cluster Ta2291; DFCI contig TC308517), a S-adenosylmethionine decarboxylase (Unigene cluster Ta53919; DFCI contig TC320440), RNase L inhibitor-like protein (Unigene cluster Ta2776; DFCI contig TC278756), GABA-receptor-associated protein (Unigene cluster Ta54963; DFCI contig TC304353). Specific primer pairs were designed for the nine genes and for the six candidate reference genes using the Beacon Designer 6 software (STRATAGENE) and imposing the following stringent criteria: T_M of 55°C ± 2°C, PCR amplicon length between 60 and 280 bp, primer length of 20 ± 2 nt, and 40 to 60% guanine-cytosine content. Primers were also designed within the 3' end region of each sequence to encompass all potential splice variants and to ensure equal RT efficiencies. The complete set of primer pairs used for qRT-PCR analyses are reported in Table 1.

Quantitative RT-PCR analyses were performed using an Mx3000PTM real time PCR system with Brillant SYBR green QPCR master mix (STRATAGENE), according to manufacturer's protocols, in 25 μl reaction volumes containing 1 μl of each fivefold diluted cDNA and 150 nM forward and reverse primers. No template and RT-minus controls were run to detect contamination, dimer formation and presence of genomic DNA. Standard curves based on five-points, corresponding to a fivefold dilution

series (1:1-1:625) from pooled cDNA, were used to compute the PCR efficiency of each primer pair. PCR efficiency (E) is given by the equation E= $(10^{[-1/m]}-1)$ x 100 [3], where m is the slope of linear regression model fitted over log-transformed data of the input cDNA concentration versus C_t values according to the linear equation y=m*log(x) + b. The thermal profile comprised three segments: 1) 95°C for 10 min; 2) 40 cycles of 30 s denaturation at 95°C, 1 min annealing at 55°C and 30 s extension at 72°C (amplification data collected at the end of each extension step); 3) dissociation curve consisting of 1 min incubation at 95°C, 30 s incubation at 55°C, a ramp up to 95°C. Two biological replicates, resulting from two different RNA extractions, RT and qRT-PCR reactions, were used in quantification analysis; three technical replicates were analysed for each biological replicate.

Only primer pairs generating a sharp peak by melting curve analysis (without unspecific products or primer-dimer artefacts) and showing efficiencies between 90 and 110% and R^2 values (coefficient of determination) calculated for standard curves higher than 0.994 were selected for expression analysis of the genes of interest (Fig. 1A) and of the candidate reference genes. The specificity of the amplicons was also checked by electrophoresis in 2% agarose gel (Fig. 1B) and by sequencing of the PCR products to confirm that their sequences corresponded to the target genes.

The number of cycles (C_t) at which the amplification-corrected normalized fluorescence (dRn) for each reaction crossed the threshold value was exported to Excel (Microsoft) for further analyses. Raw C_t values were transformed to relative quantities using the delta-C_t formula $Q=E^{\Delta Ct}$, where E is the efficiency of the primer pair used in the amplification of a particular gene and ΔC_t is the difference between the sample with the lowest C_t (highest expression) from the data set and the C_t value of the sample in question.

The expression stability of the six candidate reference genes was evaluated by the software program NormFinder (a Microsoft Excel Add-in available on the Internet) according to the author's recommendations [4]. The best combination of two genes recommended by NormFinder was that of cell division control protein and ADP-ribosylation factor, with a stability value significantly lower than that of the most stable gene (Cell division control protein) taken alone, indicating a more reliable normalization than that based on the single most stable gene. Therefore, the expression data of the nine genes of interest were normalized using the geometric average of the two reference genes Cell division control protein and ADP-ribosylation factor and their normalized relative values given as mean value + SD (Standard deviation). Standard deviations on normalized expression levels were computed according to the geNorm user manual [5].

In order to estimate the absolute copy number of cDNAs corresponding to the number of mRNAs transcribed for each of the nine PDI and PDI-like genes in different tissues and developmental stages of wheat, a standard cDNA was incorporated in each PCR run to obtain a reference curve. The cDNA standards were obtained by cloning the target sequence into the pGEM-T easy plasmid vector (Promega).

The concentration of the linearised plasmids was measured using an UV/VIS spectrophotometer Lambda 3B (Perkin Elmer). The number of copies per volume unit was calculated using the following equation [6]:

$$\text{copy number cDNA} = \frac{6.023 \times 10^{23}\ (\text{copies mol}^{-1}) \times \text{DNA amount (g)}}{\text{DNA length (bp)} \times 660\ (\text{g mol}^{-1}\ \text{bp}^{-1})}$$

The volume of the standards was first adjusted to 10^7 copies per µl and then diluted to obtain a standard series from 10^6 to 10 copies per µl, each step differing by 10 fold concentration. Each standard of the two biological replicates was analysed in duplicate and the C_t values were plotted against the logarithm of their initial template copy concentrations. Each standard curve was generated by a linear regression of the plotted points. The R^2 value, the amplification efficiency and the equation of the line [y=m*log(x) + b, where m is the slope of the line] for each of the nine standard curves of the two biological replicates are reported in Table 2. When assaying the 23 samples relative to different tissues and developmental stages of wheat, the corresponding standard series was run under the same conditions and the copy number of samples was determined by reading off the standard series with the C_t values of the samples. The calculated absolute copy numbers of the nine genes of interest were normalized using the geometric average of the relative expression of the two reference genes encoding Cell division control protein and ADP-ribosylation factor and were expressed as number of copies per µg of reverse transcribed total RNA.

References

1. Ciaffi M, Paolacci AR, D'Aloisio E, Tanzarella OA, Porceddu E: **Cloning and characterization of wheat PDI (Protein disulfide isomerase) homoeologous genes and promoter sequences.** *Gene* 2006, **366**:209-218.
2. Paolacci AR, Tanzarella OA, Porceddu E, Ciaffi M: **Identification and validation of reference genes for quantitative RT-PCR normalization in wheat.** *BMC Mol Biol* 2009, **10**:11.
3. Radonic A, Thulke S, Mackay IM, Landt O, Siegert W, Nitsche A: **Guideline to reference gene selection for quantitative real-time PCR.** *Biochem Biophys Res Commun* 2004, **313**:856-862.
4. Andersen CL, Ledet-Jensen J, Orntoft T: **Normalization of Real-Time Quantitative RT-PCR Data: A Model-Based Variance Estimation Approach to Identify Genes Suited for Normalization, Applied to Bladder and Colon Cancer Data Sets.** *Cancer Res* 2004, **64**:5245-5250.
5. geNorm manual, update July 8, 2008 [http://medgen.ugent.be/~jvdesomp/genorm/geNorm_manual.pdf]
6. Lee C, Lee S, Shin SG, Hwang S: **Real-time PCR determination of rRNA gene copy number: absolute and relative quantification assays with *Escherichia coli*.** *Appl Microbiol Biotechnol* 2008, **78**:371-376.

Table 1 . Primer pairs used in qRT-PCR analyses.

Primer pairs efficiencies and R^2 values (coefficient of determination) calculated for standard curves (5-fold dilution series from pooled cDNAs) in the two biological replicates (I and II), and characteristics of the corresponding amplicons are reported.

Gene	Forward primer	Reverse primer	Amplicon length (bp)	Amplicon Tm (°C)	Efficiencies		R^2	
					I	II	I	II
**TaPDIL1-1**	5’-CGTGGTCTTCAAATCTGG-3’	5’-GTAACCCTGGACATCAAAC-3’	187	78.0	103.3	102.1	0.999	0.997
**TaPDIL2-1**	5’-TGGTGTGGGCATTGTCAGTC-3’	5’-GGGCTTGGCACGAGGATG-3’	126	76.3	96.3	97.7	0.997	0.995
**TaPDIL3-1**	5’-GCTCCAAACTCCTAACATTGAA-3’	5’-TACTGCGTAACCGTGACCAACC-3’	166	75.3	98.5	99.8	0.999	1.000
**TaPDIL4-1**	5’-TGAGAGGCTTCATCGCTTGTTG-3’	5’-CGTGGGTCATCACCATTAAGAG-3’	122	73.3	97.3	97.6	0.997	0.997
**TaPDIL5-1**	5’-GCTACTTTCTGTTGCTGAG-3’	5’-CAAACTCGGTGATTTCGG-3’	197	77.9	98.9	100.7	0.998	0.999
**TaPDIL6-1**	5’-GAGAAAGCAGGAGAGGTGAGG-3’	5’-GGAGGCGGCAGTACAAGC-3’	151	75.8	97.9	96.1	0.998	0.996
**TaPDIL7-1**	5’-GCCAAGATGATACTCAGCCAAG-3’	5’-ACGGCGAATATCACAAGCAACC-3’	166	75.3	96.0	95.5	0.998	0.999
**TaPDIL7-2**	5’-CCTCCTCTTATTGGTGGCTGTG-3’	5’-GTGTTGGCTGGCGGTCTTCC-3’	77	72.4	98.7	97.9	0.995	0.996
**TaPDIL8-1**	5’-GCTGGTTACGCTGAGATACG-3’	5’-GATGAAGTGGGAGAATGATTTG-3’	172	74.8	100.6	102.8	0.996	0.996
**Protein AAA-superfamily ATPases**	5’-CAAATACGCCATCAGGGAGAAC-3’	5’-CGCTGCCGAAACCACGAGAC-3’	227	80.4	98.2	99.4	0.997	0.999
**ADP-ribosylation factor**	5’-GCTCTCCAACAACATTGCCAAC-3’	5’-GCTTCTGCCTGTCACATACGC-3’	165	73.7	102.8	100.8	0.997	0.996
**Rnase L inhibitor like protein**	5’-CGATTCAGAGCAGCGTATTGTT-3’	5’-AGTTGGTCGGGTCTCTTCTAAA-3’	242	73.5	98.1	99.1	0.999	0.999
**Ubiquinol-cytochrome C reductase**	5’-CCTGCCCCGTACAACCTTGAG-3’	5’-CACCGTTGCGATAGTCCTGAAA-3’	185	74.9	98.2	99.6	0.996	0.998
**S-adenosylmethionine decarboxilase**	5’-GGCTGGACAAGAAGAAGG-3’	5’-ATGGATGGTGGAGACGTC-3’	191	78.7	102.0	101.8	0.996	0.997
**GABA-receptor-associated protein**	5’-AGGAGAACAAGGACGAGGAC-3’	5’-AGGAGGCATTCAGAGCGATTG-3’	111	75.5	102.3	101.9	0.998	0.998

Table 2. Characteristics of the standard curves used for estimating the absolute copy number.

Characteristics of the standard curves used for estimating the absolute copy number of cDNAs corresponding to the transcripts of the nine PDI and PDI-like genes in different tissues and developmental stages of wheat are reported. Table shows amplification efficiencies, R2 values (coefficient of determination) and equation of the line for the standard curves of two biological replicates. For each of the nine PDI and PDI-like gene sequences a six-fold serial dilution series (from 106 to 10 copies per µl) of the recombinant pGEM-T easy plasmid was used to obtain the standard.

**Gene**	**Equation of the line**		**Efficiency**		**R^2 value**	
	I Biological replicate	II Biological replicate	I	II	I	II
TaPDIL1-1	Y= -3.397*LOG(X) + 36.91	Y= -3.356*LOG(X) + 36.56	97.8	101.1	0.997	0.999
TaPDIL2-1	Y= -3.814*LOG(X) + 40.84	Y= -3.758*LOG(X) + 40.81	92.9	94.5	0.999	0.999
TaPDIL3-1	Y= -3.701*LOG(X) + 40.77	Y= -3.701*LOG(X) + 40.82	96.0	97.2	0.997	0.996
TaPDIL4-1	Y= -3.671*LOG(X) + 39.94	Y= -3.683*LOG(X) + 39.87	97.2	94.4	0.999	0.997
TaPDIL5-1	Y= -3.577*LOG(X) + 36.49	Y= -3.524*LOG(X) + 36.32	92.4	94.9	0.999	1.000
TaPDIL6-1	Y= -3.433*LOG(X) + 37.93	Y= -3.396*LOG(X) + 37.76	95.6	97.4	0.998	0.999
TaPDIL7-1	Y= -3.356*LOG(X) + 37.26	Y= -3.346*LOG(X) + 37.16	98.6	99.2	0.999	1.000
TaPDIL7-2	Y= -3.506*LOG(X) + 39.39	Y= -3.471*LOG(X) + 39.24	92.9	94.5	0.998	1.000
TaPDIL8-1	Y= -3.464*LOG(X) + 39.76	Y= -3.448*LOG(X) + 39.68	94.4	95.0	0.996	0.995

Fig. 1. Specificity of qRT-PCR amplification.

(A) Dissociation curves of the nine wheat PDI and PDI-like genes showing single peaks (each including three technical replicates for each of 23 cDNA pools from different tissues and developmental stages). (B) Agarose gel (2%) showing the amplification of a single specific PCR product of expected size for the same genes.

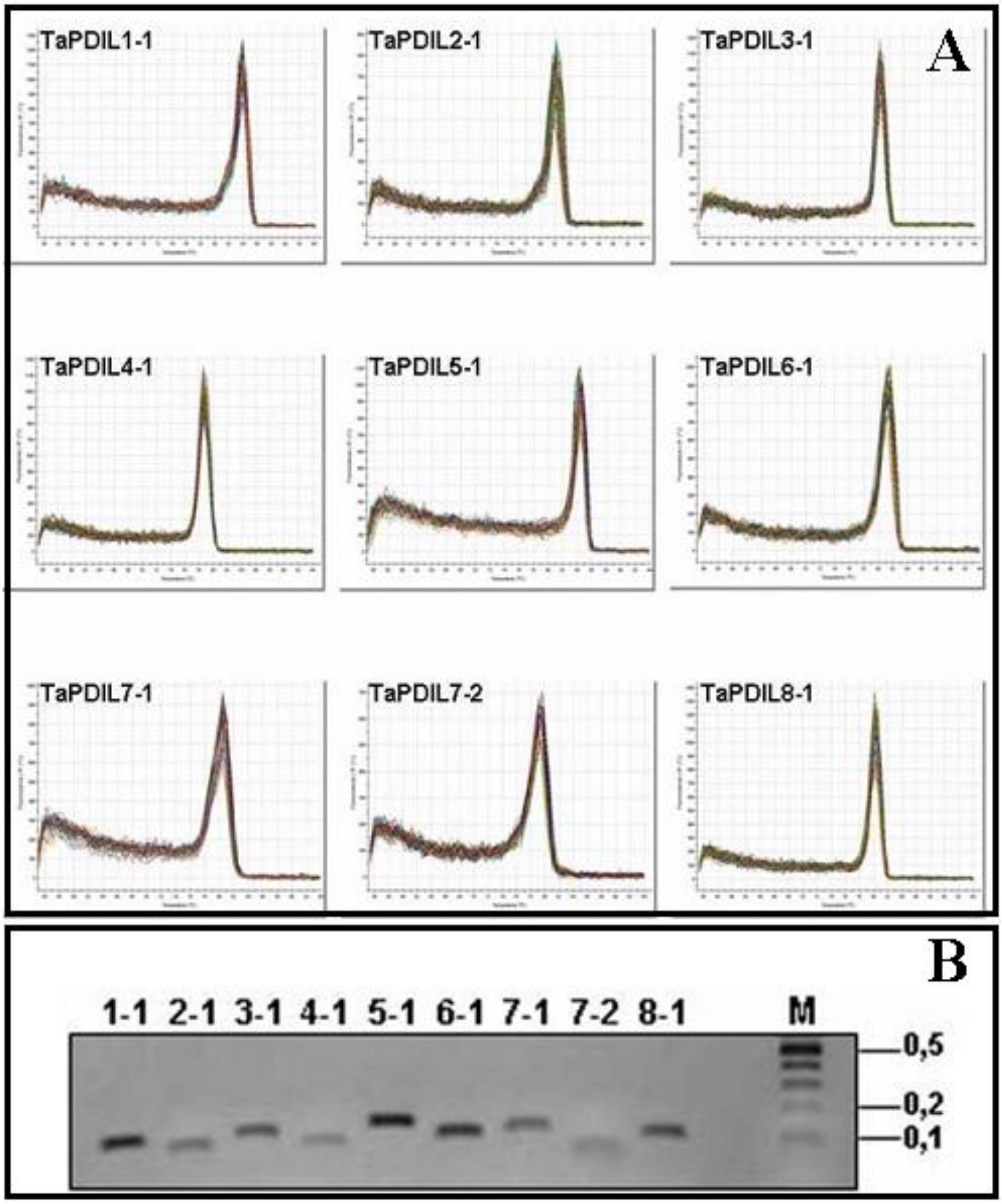

Supplement: Additional file 5 — Expression analyses by qRT-PCR of the nine wheat PDI and PDI-like genes. This additional file describes the experimental procedures for the absolute and relative quantification by qRT-PCR of the expression levels of the nine PDI and PDI-like genes. In particular it reports: a) the list of primer pairs used in qRT-PCR analyses; b) the specificity of qRT-PCR amplifications; c) the characteristics of the standard curves used for estimating the absolute copy number of cDNAs corresponding to the nine PDI and PDI-like genes; d) the method used for the normalizazion of absolute and relative data. [file 1471-2229-10-101-S5.PDF]
