# Supplementary figures and images for: The Protein Disulfide Isomerase gene family in bread wheat (T. aestivum L.)
Source: BMC Plant Biol. 2010 Jun 3;10:101. doi: 10.1186/1471-2229-10-101 (PMC3017771; doi:10.1186/1471-2229-10-101)

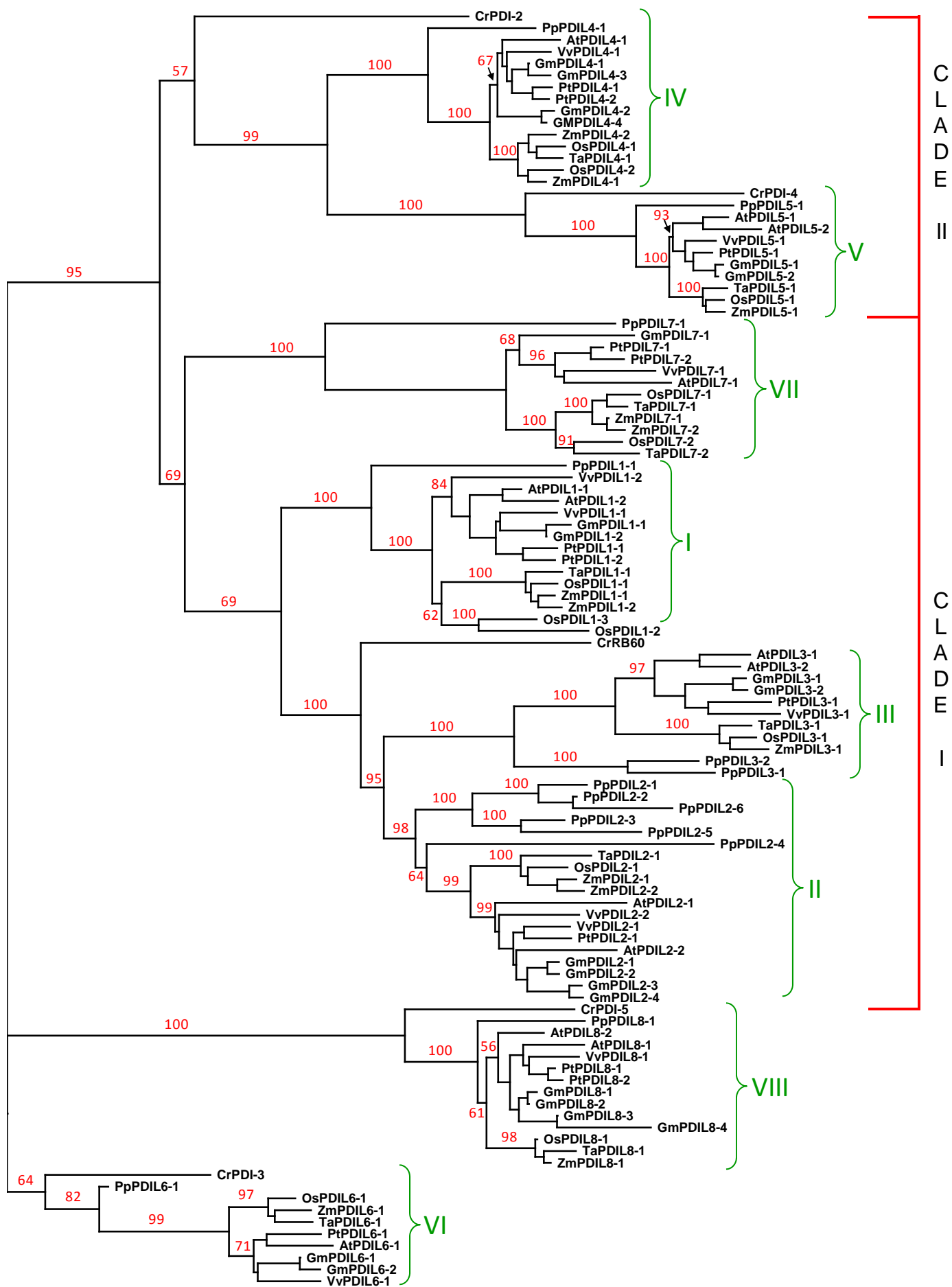

C L A D E II

C L A D E I

Supplement: Additional file 6 — Phylogenetic tree based on the deduced amino acid sequences of 108 plant PDI-like genes. The phylogenetic tree shows the relationships between the deduced amino acid sequences of the PDI and PDI-like genes of different plant species: nine of wheat, 13 of A. thaliana (At), 12 of P. trichocarpa (Pt), 10 of V. vinifera (Vv), 21 of G. max (Gm), 12 each of Z. mais (Zm) and O. sativa (Os), 14 of P. patens (Pp) and five of C. reinhardtii. Multiple alignment of the sequences was performed by ClustalX 1.83 software and the phylogenetic tree was constructed by the neighbour-joining (NJ) method and evaluated by bootstrap analysis (PHYLIP version 3.6). The numbers on the main branches indicate bootstrap percentages for 1,000 replicates. The PDI-like sequences of groups VI and VIII were used as outgroups, due to their high diversification from the other subfamilies. The two major clades (I and II) and the eight phylogenetic groups (I-VIII) indentified in the plant PDI family are highlighted with curly and square brackets, respectively. [file 1471-2229-10-101-S6.PDF]
