# Supplementary material for: The Protein Disulfide Isomerase gene family in bread wheat (T. aestivum L.)
Source: BMC Plant Biol. 2010 Jun 3;10:101. doi: 10.1186/1471-2229-10-101 (PMC3017771; doi:10.1186/1471-2229-10-101)
Supplement: Additional file 7 — Chromosome location and syntenic relationships of the PDI-like genes of wheat and rice. Chromosome location of wheat PDI-like genes determined through Southern or PCR analysis (this study), Chromosome location and position of the orthologous rice genes as well as the flanking Wheat Bin Mapped Marker mapped onto Rice Pseudomolecules http://rice.plantbiology.msu.edu/cgi-bin/gbrowse/rice/ are reported as in Release 6.1 of the MSU Rice Genome Annotation (Osa1 June 3 2009). For each Wheat Bin Mapped Marker the accession number of the corresponding probe used in the wheat deletion mapping project, wheat chromosome arms involved as well as their alignment position on the rice Pseudomolecules are also reported. Corresponding wheat chromosome bin information is not reported but can be retrieved based on probe information http://wheat.pw.usda.gov/cgi-bin/westsql/map_locus.cgi. [file 1471-2229-10-101-S7.PDF]

Wheat PDI-like gene:		Orthologous rice gene:		Wheat Bin Mapped Marker:		
**Name**	**Chromosome**	**Name (previous name)**	**Chromosome**	**Probe accession**	**Rice Chromosome**	**Wheat Chromosome arm**
TaPDIL2-1	**W6**	***OsPDIL2-1 (OsPDIL1-4)***	**R2**:8377..3514	BF474382	R2:36855..37038	6AS, 6DS, 7AL, 2BL
				BE403550	R2:53531..53806	6AS, 6BS, 6DS
				BE498351	R2:82287..82443	6AS, 6BS, 6DS
TaPDIL3-1	**W7**	***OsPDIL3-1 (OsPDIL1-5)***	**R6**:3198338..3202980	BE445587	R6:3003073..3003481	7AS, 7BS, 7DS
				BE403826	R6:3055168..3055588	7AS, 7BL, 4AL
				BF474337	R6:3263460..3263765	7AS
				BE399570	R6:3264556..3264837	7AS, 7BS
				BE499248	R6:3313435..3313757	7AS, 7BS, 7DS
TaPDIL4-1	**W1**	***OsPDIL4-1 (OsPDIL2-1)***	**R5**:3298031..3294672	BE637935	R5:3220814..3220970	1AS, 1BS, 1DS
				BE423275	R5:3232374..3232719	1AS, 7BL
				BE498820	R5:3252116..3252393	1AS, 1BS
				BF474569	R5:3346907..3347054	1AS, 1BS, 1DS
				BF292071	R5:3484508..3484734	1AS, 1BS, 1DS
TaPDIL5-1	**W5**	***OsPDIL5-1 (OsPDIL2-3)***	**R9**:16929986..16934969	BE443187	R9:16854796..16855177	5AL, 5BL, 5DL
				BE399924	R9:16857115..16857317	5BL, 6BS, 7BL, 1DL
				BE498656	R9:16924836..16925398	5AL, 5BL, 6AL, 6BL, 6DL
				**BE399897**	**R9:16932348..16932549**	**5AL, 5BL, 5DL**
				BE422943	R9:16989189..16989355	5BL, 5DL, 7DS
				BE497820	R9:17003592..17003788	5AL, 5BL
TaPDIL6-1	**W4**	***OsPDIL6-1 (OsPDIL5-1)***	**R3**:9954074..9951724	BE518424	R3:9898809..9899022	4AS, 4BL, 4DL
				BE406977	R3:9956779..9957066	1AL, 1BL, 1DL
				BE499509	R3:9973093..9973338	4AS, 4BL, 4DL
				BG314076	R3:9978902..9979103	4AS, 4BL, 4DL
TaPDIL7-1	**W2**	***OsPDIL7-1 (OsPDIL5-2)***	**R4**:21293095..21290084	BF478787	R4:21109805..21110134	2A, 2DL
				BE403177	R4:21210317..21210474	2B
				BE442794	R4:21294651..21295070	2AL, 2BL,2DL
				BG263263	R4:21513812..21514046	2AL, 2BL, 2DL, 1DS
TaPDIL7-2	**W6**	***OsPDIL7-2 (OsPDIL5-3)***	**R2**:20694520..20696830	BG263023	R2:20673459..20673610	6AS, 6BS, 6DS
				BF145399	R2:20681661..20681776	1AS, 1BS, 1DS
				**BF201426**	**R2:20694558..20694773**	**6AL,6BL,6DL**
				BE604412	R2:20757838..20757987	6AL, 6BL, 6D, 7A, 7BS, 7DS
				BE404912	R2:20796904..20797105	6AL, 6BL, 6DL
TaPDIL8-1	**W2**	***OsPDIL8-1 (OsPDIL5-4)***	**R7**:20340013..20346769	BE446243	R7:20261102..20261290	2DS, 2BS
				BE406569	R7:20304107..20304463	5AL
				BE636802	R7:20313201..20313426	2AS, 2BS, 2DS
				BF293744	R7:20454479..20454897	2BS, 1AS, 1BS
