# Supplementary material for: The Protein Disulfide Isomerase gene family in bread wheat (T. aestivum L.)
Source: BMC Plant Biol. 2010 Jun 3;10:101. doi: 10.1186/1471-2229-10-101 (PMC3017771; doi:10.1186/1471-2229-10-101)
Supplement: Additional file 9 — Expression analysis of PDI and PDI-like genes in different tissues and developing caryopses. Relative (A and C) and absolute (B and D) quantification of the expression level of nine PDI and PDI-like genes in 12 tissues and developmental stages of wheat. The 24 cDNA pools (two biological replicates, 12 plant samples) were tested in triplicate and normalized using the geometric average of the relative expression of the two reference genes encoding Cell division control protein and ADP-ribosylation factor. The relative expression levels of the nine genes were referred to that of a calibrator set to the value one, which was represented by the tissue with the lowest expression (A and C). The absolute expression levels of the nine genes were expressed as number of cDNA copies per mg of reverse transcribed total RNA (B and D). Normalized values of relative and absolute expressions of the nine genes are given as average ± SD. [file 1471-2229-10-101-S9.PDF]

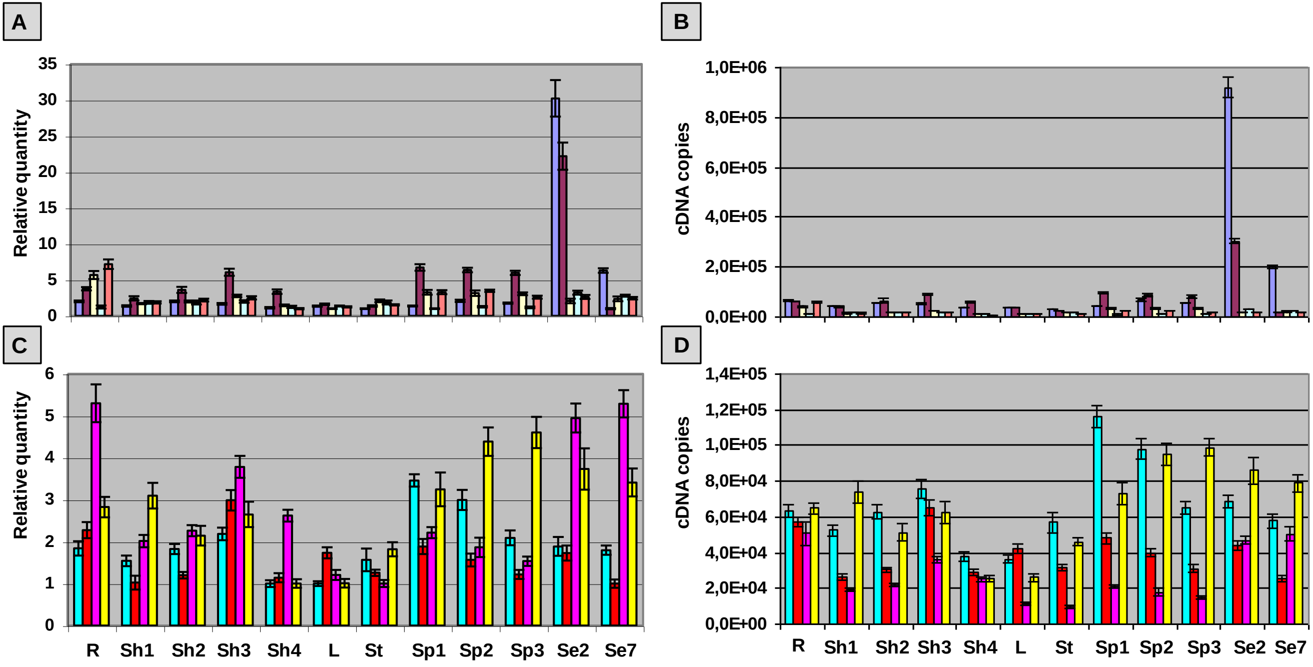

R= Roots from plants with a single shoot and three leaves unfolded (Feekes scale 1.3); Sh1= single shoots and leaves from plants at Feekes scale 1.3; Sh2= shoots at the beginning of tillering (Feekes scale 2.0); Sh3= shoots from plants after tillering (Feekes scale 3); Sh4= shoots at the beginning of erect growth (Feekes scale 4); L= flag leaves at booting stage (Feekes scale 10); St=stems at booting stage (Feekes scale 10); Sp1-3= spikes collected at intervals of 10-12 days (three developmental stages: 15-20 mm, flag leaf unfolding and heading stage); Se2 and Se7= seeds collected 10 (medium milk stage) and 38 (hard dough stage) days post anthesis (DPA).
